# Double-Decker Silsesquioxanes Self-Assembled in One-Dimensional Coordination Polymeric Nanofibers with Emission Properties

**DOI:** 10.1021/acsami.1c02510

**Published:** 2021-05-07

**Authors:** Julia Duszczak, Katarzyna Mituła, Andrea Santiago-Portillo, Loraine Soumoy, Monika Rzonsowska, Rafał Januszewski, Luca Fusaro, Carmela Aprile, Beata Dudziec

**Affiliations:** † Department of Organometallic Chemistry, Faculty of Chemistry, Centre for Advanced Technologies, Adam Mickiewicz University in Poznan, Uniwersytetu Poznanskiego 8 and 10, 61-614 Poznan, Poland; ‡ Department of Chemistry, University of Namur, Rue de Bruxelles 61, 5000 Namur, Belgium; § Department of Chemistry and Technology of Silicon Compounds, Faculty of Chemistry, Centre for Advanced Technologies, Adam Mickiewicz University in Poznan, Uniwersytetu Poznanskiego 8 and 10, 61-614 Poznan, Poland

**Keywords:** double-decker silsesquioxanes, silylative coupling reaction, sonogashira reaction, hybrid materials, nanofibers, light emitting materials, *E*−*Z* isomerization

## Abstract

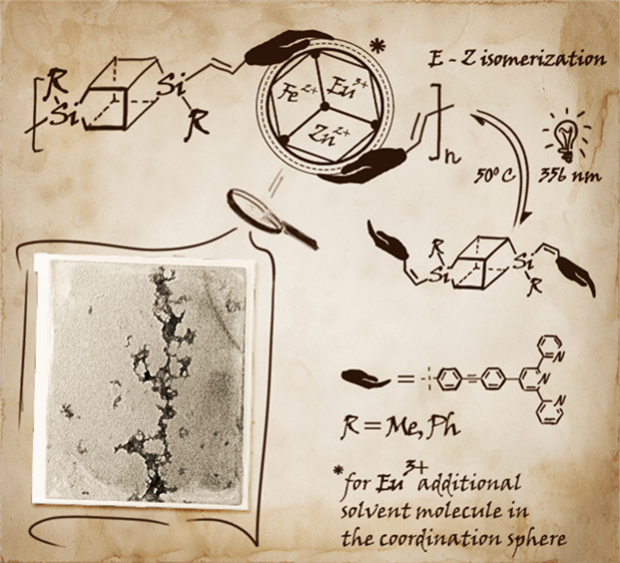

The urgent needs for photoactive materials in industry drive fast evolution of synthetic procedures in many branches of chemistry, including the chemistry of silsesquioxanes. Here, we disclose an effective protocol for the synthesis of novel double-decker silsesquioxanes decorated with two (styrylethynylphenyl)terpyridine moieties (**DDSQ_Ta-b**). The synthesis strategy involves a series of silylative and Sonogashira coupling reactions and is reported for the first time. **DDSQ_Ta-b** were employed as nanocage ligands to promote self-assembly in the presence of transition metals (TM), i.e., Zn^2+^, Fe^2+^, and Eu^3+^ ions, to form one-dimensional (1D) coordination polymeric nanofibers. Additionally, ultraviolet-promoted and reversible *E*–*Z* isomerization of the C=C bond within the ligand structures may be exploited to tune their emission properties. These findings render such complexes promising candidates for applications in materials chemistry. This is the first example of 1D coordination polymers bearing DDSQ-based nodes with TM ions.

## Introduction

Oligomeric silsesquioxanes (**SQs**) constitute a broad family of organosilica compounds known for their various architectures from random, ladder, and incompletely condensed to well-defined cages.^1,2^ Their uniqueness results from the presence of an inorganic siloxane Si–O–Si core and tunable functional organic coronae, which classify them as hybrid systems. These compounds are attracting increasing attention due to their exclusive properties derived from chemically and thermally robust organic–inorganic frameworks and tailor-made three-dimensional structures. They display great potential in the formation and modification of polymeric systems, affecting their physicochemical properties, e.g., enhanced thermal and mechanical stability, oxidation resistance and nonflammability, solubility, good dielectric properties, etc. All these features are of interest for multiple applications.^3,4^ To date, scientific interest in silsesquioxanes was fixed mostly on functionalized cubic T_8_ (mono- and octasubstituted) derivatives.^2,4−7^ However, since the discovery of a new class of the so-called double-decker (DDSQ) type of silsesquioxane by Yoshida et al., it has attracted attention in the world of organosilicon compounds.^8−10^ Studies on the synthesis of double-decker silsesquioxanes involve utility of the “closed” core derivatives, described as D_2_T_8_ and the “open” core, i.e., M_4_T_8_, and refer to di- and tetrafunctionalized DDSQ compounds, respectively. This in turn affects the application of these systems in the formation of molecular and macromolecular DDSQ-based systems.^11−21^ However, there have been a limited number of scientific reports concerning double-decker silsesquioxanes in comparison with cubic T_8_ structures. This bottom-up approach toward formation of desired silsesquioxane derivatives has gained wide interest. It is due to the ease of functionalization of both cubic T_8_ and DDSQ moieties with a variety of substituents (i.e., hydrosilylation, cross-metathesis, and silylative and Heck coupling), which can be utilized for the design and preparation of precisely controlled nanomaterials.^12,22,23^

Owing to the appealing features of silsesquioxanes, combining the properties of silica in an inorganic core and easily tunable organic moieties anchored to it, various attempts to use them as a specific ligand in transition metal (TM) coordination compounds have also been explored. There are some reports on the application of mono- and octafunctionalized silsesquioxanes with a specific functional group, playing the role of a ligand, mainly bi- or tridentate, e.g., diketones,^24,25^ carboxylic acids,^26,27^ 8-hydroxyquionoline,^28,29^ and terpyridine derivatives,^30−35^ but also monodentate, as phosphines^36^ or amines.^37^ The metals that may be used as coordination centers are rather restricted to transition metals, e.g., mainly Ru, Pd, and Fe or Cu, Pt, and Zn but also Ln (Eu and Tb).

The obtained compounds form either molecular or macromolecular 3D coordination systems (coordination polymers) exhibiting attractive physicochemical features, e.g., large shifts in absorption–emission spectra (incl. metal-to-ligand charge transfer (MLCT) bands) or photoelectrochemical properties, and may be used in the preparation of photoactive luminescent materials and devices^27,31,32,38−40^ or as effective catalysts,^37,41^ etc. Interestingly, for the double-decker-based coordination systems, the number of reports concerning their synthesis, characterization, and application is quite limited.^42,43^ Studies by Yam et al. refer to the synthesis of “closed” DDSQ structures bearing two terpyridine (Tpy)-functionalized substituents with the consequent possible presence of stereoisomers and their further use in the formation of Pt^II^-based coordination systems.^43^ The obtained molecular compounds exhibited interesting self-association via Pt···Pt interactions. On the other hand, the work of Kucuk et al. presents the “open” DDSQ architecture with two Tpy derivatives attached to it that form a coordination macromolecular system with Ru^II^ ions.^42^ These studies refer to the interesting photo- and electrochemical properties as well as the assembling ability of [Ru(Tpy)_2_]^2+^ moieties in the DDSQ-based frameworks.

Encouraged by these reports, here, we present a novel synthetic strategy to obtain the “closed” type of DDSQ with two (styrylethynylphenyl)terpyridine anchored to the opposite corners. To the best of our knowledge, this represents the first report on the use of consecutive silylative coupling and Sonogashira reactions in the chemistry of double-decker silsesquioxanes. The resulting products were obtained with high overall yields (up to 72%) and selectivity, and their thermal stabilities were also verified. Moreover, the coordinating abilities of the novel DDSQ were tested by selecting three different TM ions, i.e., Fe^2+^, Zn^2+^, and Eu^3+^. To investigate the photochemical features of the **TM@DDSQ_Ta-b** complexes, they were thoroughly characterized via UV–vis and photoluminescence (PL) spectroscopy. Interestingly, the construction of linear 1D coordination polymers encompassing metal ion nodes and functionalized DDSQ spacers that formed nanofibers was observed and confirmed by TEM analysis.

Due to the presence of both functionalized silsesquioxane fragment as well as TM ions, the obtained compounds may have potential applications as sensors and light-emitting components for luminescent devices,^44−47^ photoswitchable materials,^48^ or functional hybrid polymers.^49,50^

## Experimental Section

### Materials

The chemicals were purchased from the following sources: Sigma-Aldrich for toluene, 4-bromostyrene, triethylamine, hexane, tetrahydrofuran, methanol, dichloromethane, Pd(PPh_3_)_4_, CuI, Fe(OTf)_2_, Zn(OTf)_2_, Eu(OTf)_3_, and silica gel 60; TCI for anhydrous THF; and Fischer Chemical for absolute EtOH and HN(iPr)_2_. The following double-decker silsesquioxanes: DDSQ-2(MeSiVi), DDSQ-2(PhSiVi),^51^ Tpy-T,^52^ and [RuH(CO)Cl(PCy_3_)_2_]^53^ were prepared according to the literature procedure. All solvents were dried over CaH_2_ prior to use and stored under argon over 4 Å molecular sieves. All liquid substrates were also dried and degassed by bulb-to-bulb distillation. All syntheses were conducted under an argon or nitrogen atmosphere using standard Schlenk line and vacuum techniques.

### Measurements

#### Nuclear Magnetic Resonance (NMR)

^1^H, ^13^C, and ^29^Si nuclear magnetic resonance (NMR) measurements for **DDSQa-b** were performed on Bruker 400 MHz or 300 MHz spectrometers using CDCl_3_ as a solvent. Chemical shifts are reported in ppm with reference to the residual solvent (CHCl_3_) peaks for ^1^H and ^13^C and to TMS for ^29^Si.

Quantitative ^1^H NMR experiments for **DDSQ_Ta-b** were performed at 25 °C on a Varian VNMRS spectrometer operating at 9.4 T (400 MHz for ^1^H) equipped with a 5 mm broadband probe, using the following acquisition parameters: a relaxation delay of 8.0 s, an acquisition time of 2.0 s, an excitation pulse of 90°, and 32 transients.

#### Solid-State Nuclear Magnetic Resonance (ssNMR)

Solid-state ^13^C and ^29^Si NMR spectra were recorded at room temperature on a Bruker Avance-500 spectrometer operating at 11.7 T (125.7 MHz for ^13^C and 99.3 MHz for ^29^Si) using a 4.0 mm probe and spinning frequencies of 8 and 10 kHz.

#### Matrix-Assisted Ultraviolet Laser Desorption/Ionization Time-of-Flight Mass Spectroscopy (MALDI-TOF-MS)

Matrix-assisted laser desorption/ionization time-of-flight (MALDI-TOF-MS) mass spectra were recorded on an UltrafleXtreme mass spectrometer (Bruker Daltonics), equipped with a SmartBeam II laser (355 nm) in the 500–4000 *m*/*z* range. 2,5-Dihydroxybenzoic acid (DHB, Bruker Daltonics, Bremen, Germany) served as a matrix and was prepared in a TA30 solvent (30:70 v/v acetonitrile/0.1% TFA in water) at 20 mg/mL concentration. Studied samples were dissolved in dichloromethane (2 mg/mL) and then mixed in a ratio of 1:1 v/v with matrix solution. Matrix/sample mixtures (1 μL) were spotted onto the MALDI target and air-dried. Mass spectra were measured in reflection mode. The data were analyzed using the software provided with the Ultraflex instrument—FlexAnalysis (version 3.4). Mass calibration (cubic calibration based on five to seven points) was performed using external standards (Peptide Calibration Standard).

#### FT-IR Spectroscopy

Fourier transform infrared (FT-IR) spectra were recorded on a Nicolet iS5 (Thermo Scientific) spectrophotometer equipped with a diamond ATR unit. In all cases, 16 scans at a resolution of 2 cm^–1^ were collected, to record the spectra in a range of 4000–650 cm^–1^.

#### Elemental Analysis

Combustion chemical analysis (C, H, and N) was performed on a PerkinElmer 2400 Series 2 analyzer.

#### TGA

TGA analyses were performed using a TGA/DSC 1 Mettler Toledo thermal gravimetric analyzer. The measurements were conducted in a nitrogen and air atmosphere (flow of 60 mL/min), from ambient temperature to 1000 °C at a heating rate of 10 °C/min. The temperature of initial degradation (*T*_d_) was taken as the onset temperature at which 5 wt % mass loss occurs.

#### TEM

Transmission electron microscopy images were recorded with a PHILIPS TECNAI 10 instrument at 80 kV.

#### SEM

Scanning electron microscopy images were recorded with a JEOL 7500F coupled with an EDX.

#### UV–Vis and Fluorescence Measurements

UV–vis measurements were performed on a Cary 5000 spectrophotometer (Varian) and fluorescence measurements on a Cary Eclipse instrument (Agilent Technologies). The measurements were taken using 10 mm Suprasil quartz cuvettes from Hellma Analytics.

#### X-ray Analysis

X-ray diffraction data were collected at 100(1) K, by the ω-scan technique on an Agilent Technologies four-circle Xcalibur diffractometer (Eos detector) with graphite monochromatized Mo Kα radiation (λ = 0.71073 Å). The data were corrected for Lorentz polarization and absorption effects.^54^ Using Olex2,^55^ the structure was solved with the ShelXT^56^ structure solution program using Intrinsic Phasing and refined with the ShelXL^57^ refinement package using least-squares minimization. All nonhydrogen atoms were refined anisotropically, and all hydrogen atoms were placed in the calculated positions and refined as a “riding model” with the isotropic displacement parameters set at 1.2 times the Ueq value for appropriate nonhydrogen atoms.

## Results and Discussion

**Scheme 1:**
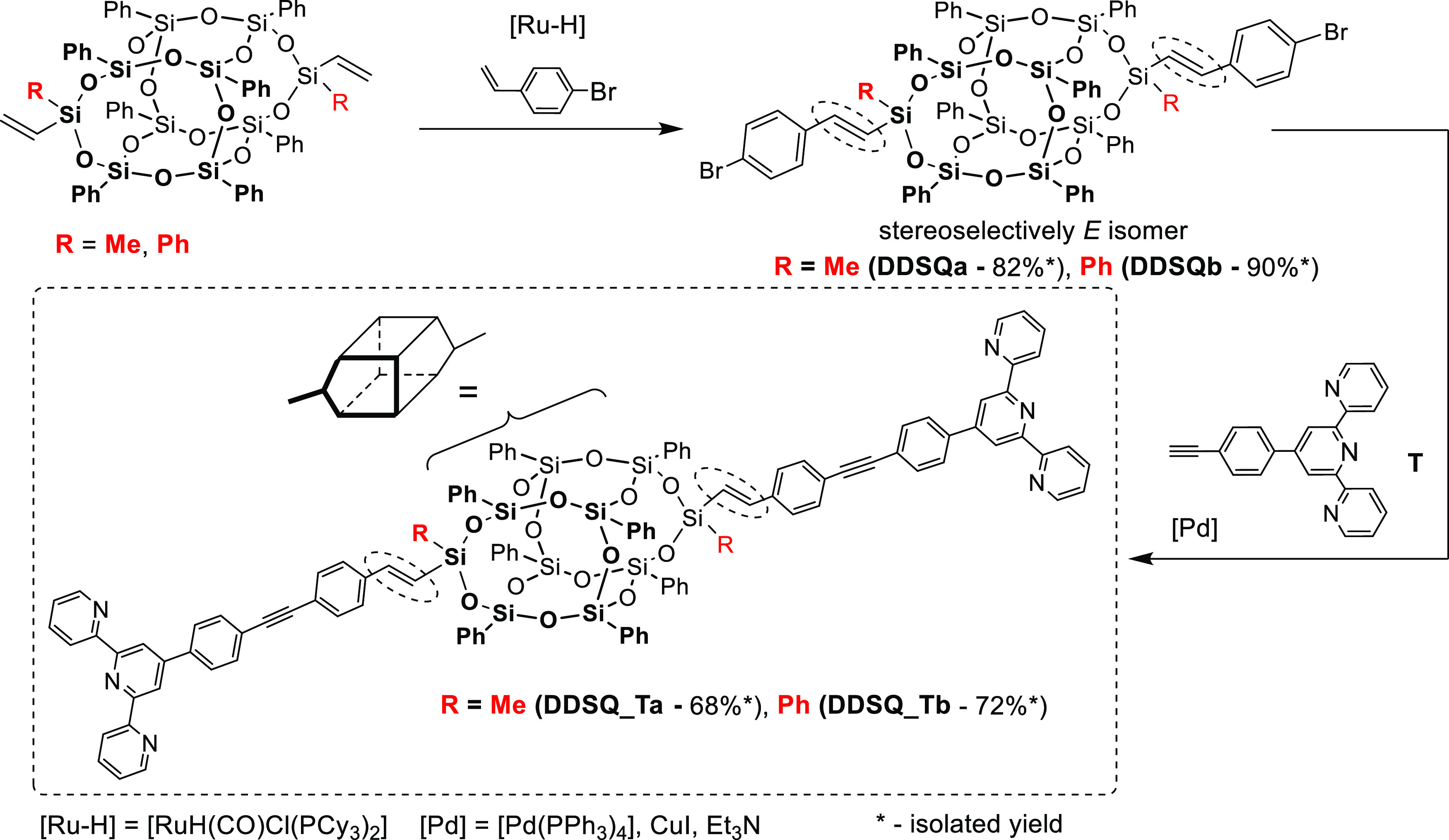
Schematic Depiction for the Synthesis of **DDSQa-b** and **DDSQ_Ta-b** via Consecutive Silylative Coupling and Sonogashira Reaction

Double-decker-shaped silsesquioxanes possess some structural differences from the cubic, octafunctional T_8_ derivatives that offer additional advantages in the formation of coordination complexes, e.g., easy purification, relatively less rigidness, and reduced self-aggregation tendencies.^42^ The synthetic protocol applied to obtain DDSQs decorated with two (styrylethynylphenyl)terpyridine moieties was based on a silylative coupling reaction followed by Sonogashira coupling. While the Sonogashira reaction is known in the chemistry of cubic T_8_ silsesquioxanes,^58−60^ this is a novel synthetic approach in the chemistry of DDSQ systems. Laine et al. exploited Sonogashira reaction for modification of octa(iodo)- and octa(bromophenyl)silsesquioxanes in the presence of Pd(PPh_3_)_4_/CuI or Pd_2_(dba)_3_ with Pd(Pt-Bu_3_)_2_ in 1,4-dioxane. These reactions were conducted for 24–48 h at 60 °C (iodophenyl derivatives) or RT (bromophenyl derivatives).^58^ Okubo et al. used Sonogashira coupling for modification of octa(bromostyryl)silsesquioxanes using an analogous Pd(PPh_3_)_4_/CuI catalytic system or Na_2_PdCl_4_ and [(t-Bu)_3_PH]BF_4_/CuI in THF at 70 °C for 48 h.^60^ Ervithayasuporn and co-workers applied this process to post modification of *p*-phenylene-ethynylenes with monosubstituted cubic T_8_ SQs as pendant groups using (PPh_3_)_2_PdCl_2_/CuI in THF at RT for 24 h.^59^ To the best of our knowledge, these are the only few examples of Sonogashira reaction used for the modification of cubic T_8_ silsesquioxanes. Thus, a test of its use in the case of DDSQ silsesquioxanes was performed here. To functionalize DDSQs with two (styrylethynylphenyl)terpyridine moieties (**DDSQ_Ta** and **DDSQ_Tb**), an innovative reaction strategy for the functionalization of DDSQ systems was elaborated (Scheme 1).

First, the di(*p*-bromostyryl)-substituted DDSQs were obtained by selective silylative coupling reactions (**DDSQa-b**). Interestingly, for the **DDSQa** with the Me substituent at the (D)Si atom, the mixture of cis and trans geometric isomers was obtained (core resonance lines at ^29^Si NMR: δ = −78.28, −79.31 (cis), −79.55 (trans), and −79.77 (cis) (−Si–C_6_H_5_)), while in the case of **DDSQb** with the Ph substituent at the (D)Si atom, the exclusive formation of the trans isomer was proven (core resonance lines at ^29^Si NMR: δ = −77.93 and −79.41 (trans) (−Si–C_6_H_5_)). For details, see Figures S1–S7. 4′-(4-Ethylnylphenyl)-[2,2′:6,2″]terpyridine (**T**) was then synthesized, and the structure was confirmed via ^1^H and ^13^C NMR (Figures S8 and S9).

The next step was to exploit **DDSQa-b** in the Sonogashira reaction with (ethynylphenyl)terpyridine (**T**) in the presence of Pd(PPh_3_)_4_/CuI as a catalytic system in THF at 70 °C for 24 h. For both compounds, the reaction was efficient and enabled the synthesis of designed DDSQ-based systems with two (styrylethynylphenyl)terpyridine groups anchored to the Si–O–Si core. All the obtained products are air-stable solids and can be synthesized on a multigram scale (see Table S1 in the Supporting Information). **DDSQa** and **DDSQb** are soluble in organic solvents like dichloromethane (DCM), CHCl_3_, tetrahydrofuran (THF), and toluene but not in methanol, MeCN, and hexane. They were isolated and characterized using ^1^H, ^13^C, and ^29^Si NMR and FT-IR (see Supporting Information, Figures S10–S15), and their thermal stability was verified via thermogravimetric analysis (TGA). It should be noted that due to the low solubility of **DDSQ_Ta** and **DDSQ_Tb**, ^13^C and ^29^Si NMR was performed in solid-state (Figures S11 and S12 and S14 and S15). ^29^Si cross polarization (CP) NMR spectra of **DDSQ_Ta** and **DDSQ_Tb** were recorded by spinning the sample at the magic angle (MAS).

As seen in the ^29^Si NMR spectra in the Supporting Information, two signals, centered at −35.99 and −84.94 ppm in the case of **DDSQ_Ta** and at −50.71 and −84.21 ppm in the case of **DDSQ_Tb**, can be discerned. The first ones are assigned to the Si(OSi)_2_R_2_ moieties (D^2^), while the second and more shielded signals are assigned to the Si(OSi)_3_R moieties (T^3^). The integrated areas of these signals are in agreement with the expected values.

**Figure 1:**
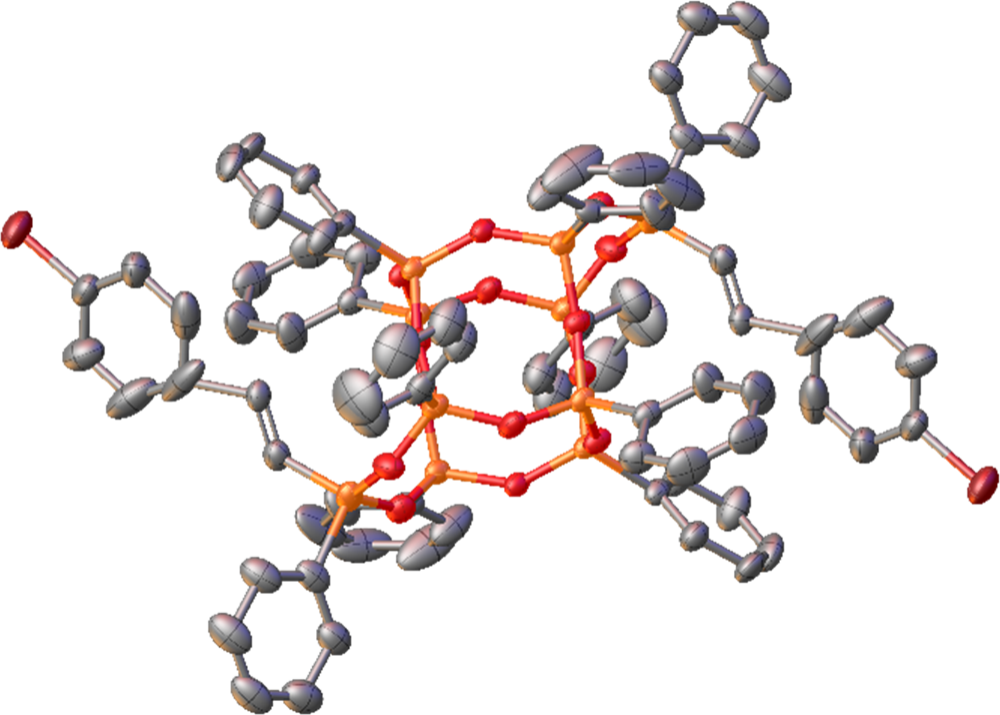
Perspective view of the molecule **DDSQb**. Ellipsoids are shown at the 33% probability level. Hydrogen atoms are omitted for clarity.

The crystal structure of **DDSQb** was also obtained. The perspective view of the **DDSQb** molecule is presented in Figure 1.

### Thermogravimetric Analysis (TGA)

To determine the effect of the DDSQ modification on thermal stability, the synthesized organosilicon derivatives (**DDSQa-b** and **DDSQ_Ta-b**) as well as the 4′-(4-ethynylphenyl)-2,2′-terpyridine (**T**) solids were investigated via thermogravimetric analysis in a nitrogen and air. The results are summarized in Tables 1 and 2 and presented in Figures S16 and S17 (see the Supporting Information). TGA revealed the significant influence of the chemical structure of the compounds on their thermal stability. **DDSQ_Ta** exhibits similar thermal stability to the precursor (**DDSQa**). On the other hand, **DDSQ_Tb** revealed lower thermal resistance than the corresponding **DDSQb**, which was manifested by a decrease in the initial degradation temperatures (*T*_d_^5%^). Moreover, *T*_max_ (the temperature of the maximum weight loss rate) values determined for **DDSQ_Ta-b** significantly decreased when compared to **DDSQa-b**. This effect can be explained by the increased content of the organic phase, which is responsible for thermo-oxidative stability. On the other hand, it is commonly known that the halogenated compounds are very active in the gas phase and act as free radical scavengers, which prevent further thermal degradation.^61^ It is reflected in other papers describing synthesis and thermal properties of new halogen-containing SQ derivatives. The results published by Laine and co-workers clearly indicate that the thermal stability increases with the increasing number of bromine atoms covalently bonded to the silsesquioxane core.^62^

**Table 1:** Thermal Properties of Substrates (**DDSQa-b**) and Obtained Compounds (**DDSQ_Ta-b**) Measured in N_2_

	mass loss temperature [°C]			
prod. abbreviation	*T*_d_^5%^	*T*_d_^10%^	*T*_max_ [°C]	residue at 1000 °C [%]
**DDSQa**	387	413	454, 576	55
**DDSQb**	433	459	463, 568	64
**DDSQ_Ta**	389	432	436, 614	67
**DDSQ_Tb**	372	436	338, 418, 471, 638	70
**T**	456	523	440, 491, 556, 690	35

**Table 2:** Thermal Properties of Substrates (**DDSQa-b**) and Obtained Compounds (**DDSQ_Ta-b**) Measured in Air

	mass loss temperature [°C]			
prod. abbreviation	*T*_d_^5%^	*T*_d_^10%^	*T*_max_ [°C]	residue at 1000 °C [%]
**DDSQa**	381	419	437, 622	36
**DDSQb**	404	446	448, 627	37
**DDSQ_Ta**	378	456	381, 655	26
**DDSQ_Tb**	367	448	374, 625	23
**T**	465	506	473, 639	0

Moreover, Wada et al. published the Pd-catalyzed arylation of open-cage silsesquioxanes. Their results confirmed that the presence of the bromophenyl unit significantly increases thermal stability when compared to phenyl counterparts.^63^

It should be also noted that **DDSQb**-containing Si–Ph groups revealed higher thermal stability than the methyl derivative (**DDSQa**), consistent with the literature.^64,65^ The opposite order was observed after the DDSQ functionalization through Sonogashira coupling; nevertheless, the differences in the *T*_d_^5%^ values did not turn out to be significant for **DDSQ_Ta-b** as was observed for **DDSQa-b**. Most of the samples exhibited a bimodal decomposition mechanism; however, a multimodal degradation was observed for **DDSQ_Tb** and **T** in a nitrogen atmosphere, manifested by the appearance of two main peaks at DTG curves. DTG curves can be found in the Supporting Information (see Figures S18–S27). Furthermore, we found that the *T*_d_^5%^ temperatures determined for **DDSQa-b** and **DDSQ_Ta-b** as well as residues after the measurements were higher for experiments performed in the inert atmosphere. Surprisingly, the highest thermal resistance was observed for **T**. However, a detailed analysis of the recorded TGA/DSC curves revealed that the **T** melts at 194 °C followed by an exothermic reaction (Figures S28 and S29 in the Supporting Information), which can be considered as an alkyne polymerization/cross-linking that prevents further decomposition. This may suggest higher thermal stability resulting from the presence of the cross-linked product rather than monomer **T**. This is in agreement with the literature describing that the presence of the cross-linked alkynes in the polymer matrix positively affects its thermal properties.^66−68^

### Photophysical Investigation

Once the synthesis of the novel DDSQs functionalized with terpyridine moieties was successfully achieved, the photophysical properties of both compounds were explored via UV–visible absorption and emission spectroscopies. In our preliminary studies, we performed photophysical investigation of the starting materials **DDSQa** and **DDSQb**, and their behavior was compared with the corresponding **DDSQ_Ta** and **DDSQ_Tb** compounds (see Figures S30 and S31 in the Supporting Information). The amount of reports of photophysical properties of DDSQs is still restricted to selected derivatives (e.g., vinyl- or styryl-).^69−71^

As it can be observed in Figure S30, **DDSQa** presents one broad absorption band centered at c.a. 280 nm due to the presence of the styryl moiety linked to the **DDSQa-b** structure.^72^ As expected, this sample did not display any emission. In the case of the **DDSQ_Ta** compound, two absorption bands centered at 290 and 330 nm were clearly observed. These contributions are related to the heterocyclic moieties, and they are very similar to those reported for terpyridine.^31,35^ As expected, a band centered at 386 nm appeared in the emission spectra, thus proving that the DDSQ scaffold does not negatively interact with the terpyridine moieties causing some strong deactivation with a total quenching of the emission. The broadness of this band, which extends till 500 nm, could be attributed to the formation of intermolecular excimers in the solution.^31,35,73,74^ This broadness observed also in more concentrated samples is reduced but does not disappear completely in the concentration range reported here. Very similar behavior was observed for the **DDSQb/DDSQ_Tb** compounds. Figure S31 shows the UV–vis and fluorescence spectra of the compounds **DDSQb** and **DDSQ_Tb**. Both samples **DDSQ_Ta** and **DDSQ_Tb** display a blue emission under a UV lamp detected by the naked eyes, thus suggesting the absence of direct intramolecular interaction between terpyridine units with consequent strong self-quenching phenomena leading to the disappearance of the emission band. This intramolecular interaction is most probably prevented by the *E* configuration of the double bond as well as by the localization of the terpyridine units at the two opposite corners of the DDSQ core (Figures S30–S31).^31^

All previous findings indicate that the DDSQ-terpyridine compounds are promising candidates for the formation of novel self-assembled structures based on metal to ligand interactions. To have a deep understanding of the complexing properties of the **DDSQ_Ta** and **DDSQ_Tb** ligands, UV–vis and emission titration experiments were performed by employing two metals able to form octahedral complexes (Fe^2+^ and Zn^2+^) and an additional one from the family of lanthanides (Eu^3+^), which can display an extended coordination shell.^24^ Moreover, the employment of the Eu^3+^ as a metal cation may present additional advantages linked to intense line-like emission bands at high wavelengths (centered at 580, 591, 617, 650, and 698 nm).

**Figure 2:**
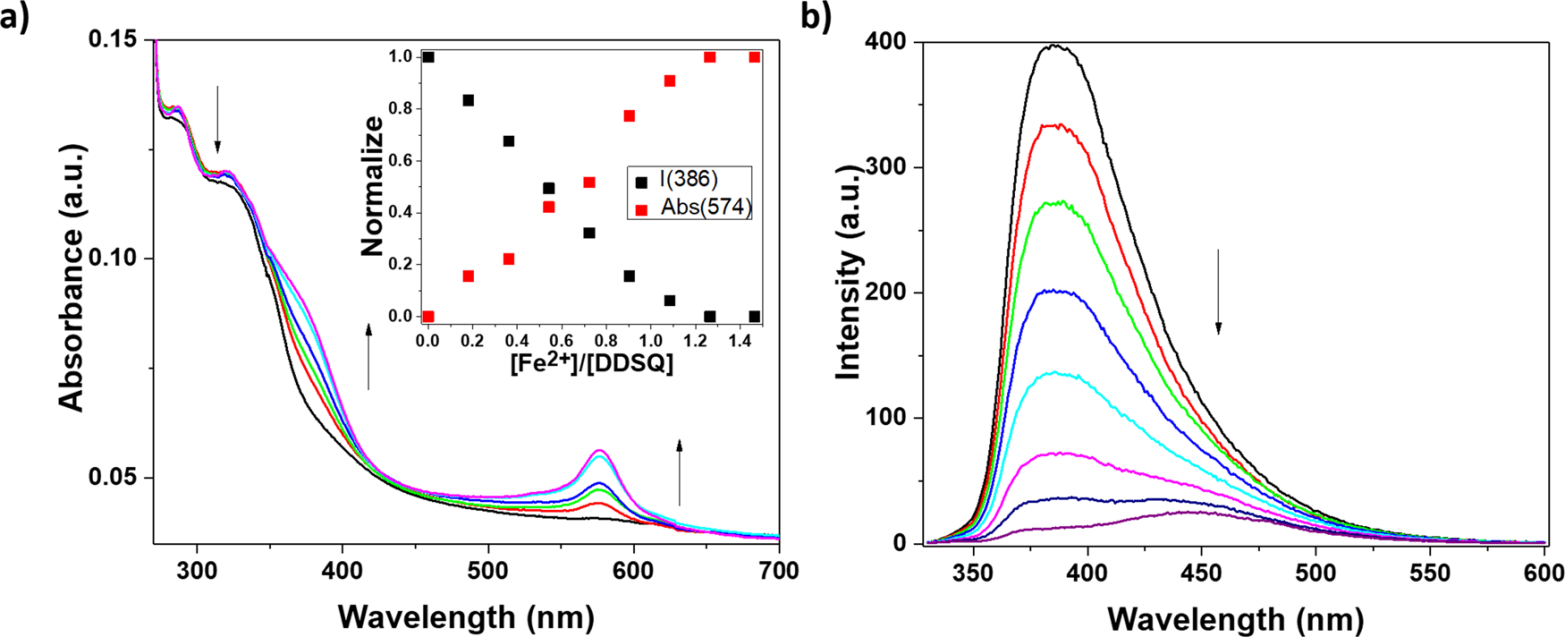
(a) UV–vis absorption spectra of the **DDSQ_Ta** compound and the normalized absorption changes at 574 nm (red squares) and the normalized emission intensity changes at 386 nm (black squares). (b) Emission spectra of the **DDSQ_Ta** sample in CH_2_Cl_2_ (1 × 10^–6^ M) upon titration with Fe(OTf)_2_ in EtOH (3.63 × 10^–4^ M). λ_ex_ = 310 nm, OD = 0.13, and slits = 5 nm.

It is known^31,35^ that metal to ligand stoichiometry could be evaluated via titration experiments followed via both ^1^H NMR and UV–vis/fluorescence spectroscopies. However, as mentioned previously, the extremely low solubility of the **DDSQ_Ta** and **DDSQ_Tb** ligands hampers the possible investigation via ^1^H NMR. Hence, UV–vis was selected as a technique of choice, and the titration experiments were initially performed by employing Fe^2+^ and Zn^2+^ species. Upon the addition of an increasing amount of iron(II) trifluoromethanesulfonate, Fe(OTf)_2_, to the **DDSQ_Ta** ligand, a new band centered at 574 nm, typical of the MLCT, appears in the UV–visible spectra (Figure 2).

Moreover, a redshift of the terpyridine absorption band at 293 nm with an isosbestic point at 310 nm was observed. Upon excitation at the isosbestic point, a complete quenching of the emission band at 386 nm was detected. This behavior is not surprising and was previously observed in the presence of other terpyridine-based ligands.^31,35^

Importantly, the plot of the normalized variation of the absorption and emission bands (centered respectively at 574 at 386 nm) indicates that a plateau is reached after the addition of 1 equiv of Fe(OTf)_2_. These results prove that 1 equiv of the metal is required to coordinate the DDSQ-based ligand forming a **Fe@DDSQ_Ta** complex with 1:1 metal to ligand stoichiometry. Since each DDSQ ligand possesses two coordinating terpyridine units on the two opposite corners, the formation of self-assembled coordinating polymeric chains could be envisaged.

Similar behavior was observed in the presence of the **DDSQ_Tb** ligand (see Figure S32 in the Supporting Information) suggesting formation of a complex **Fe@DDSQ_Tb**. Figure S33 shows the UV–vis spectra of Fe(OTf)_2_.

**Figure 3:**
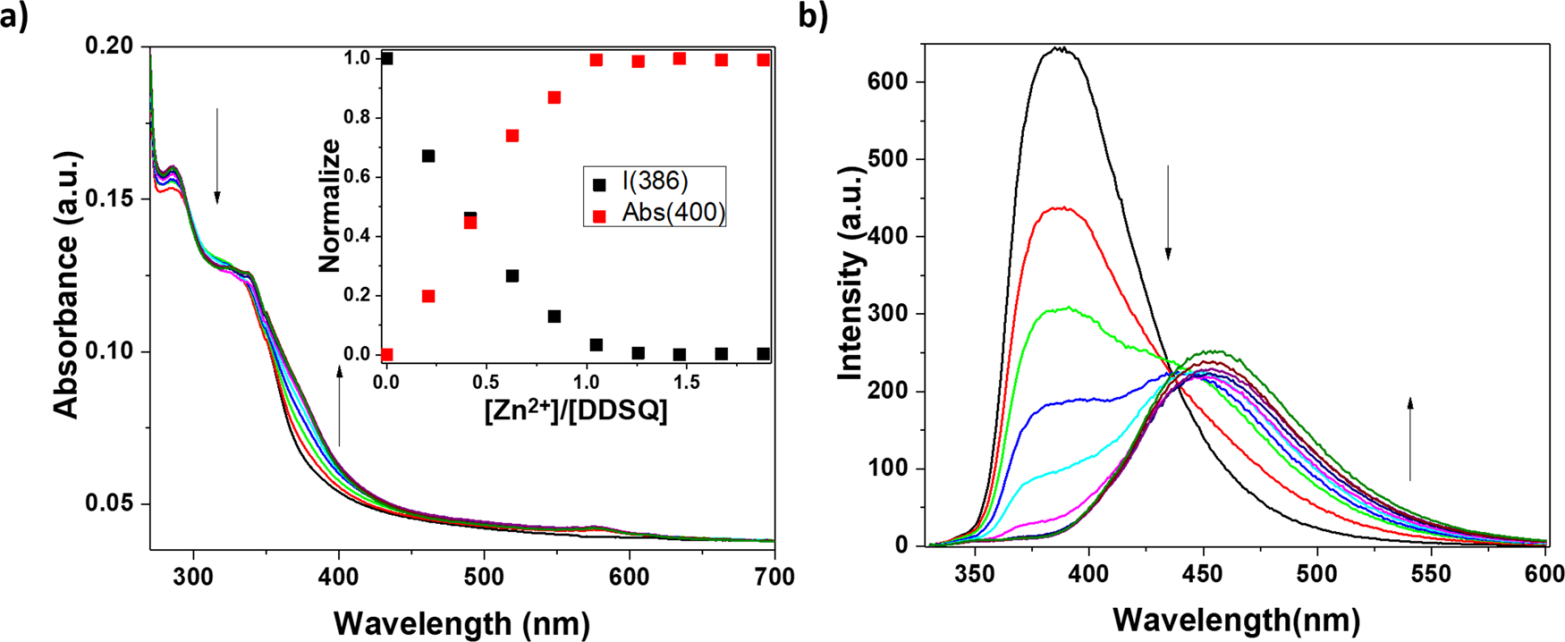
(a) UV–vis absorption spectra of the **DDSQ_Ta** sample in CH_2_Cl_2_ (1 × 10^–6^ M) upon titration with Zn(OTf)_2_ in EtOH (4.18 × 10^–4^ M). The inset shows the normalized absorption changes at 400 nm (red squares) and the normalized emission intensity changes at 386 nm (black squares). (b) Emission spectra of the **DDSQ_Ta** sample in CH_2_Cl_2_ (1 × 10^–6^ M) upon titration with Zn(OTf)_2_ in EtOH (4.18 × 10^–4^ M). λ_ex_ = 310 nm, OD = 0.13, and slits = 5 nm.

Analogous experiments were performed using zinc trifluoromethanesulfonate (Zn(OTf)_2_). Figure 3 shows the variations of the UV–visible absorption bands of the ligand **DDSQ_Ta** as a function of the addition of increasing amounts of metal cations. The results of this titration were similar to previously observed in the presence of Fe(OTf)_2_. However, in this case, upon excitation at the isosbestic point, a new contribution at c.a. 470 nm was detected. This novel band can be ascribed to the formation of a Zn@terpyridine complex (**Zn@DDSQ_Ta**). In line with the previous observations, the plot of the normalized variation of the intensity of absorption and emission bands (centered respectively at 400 and 386 ppm) suggests the formation of a complex with a 1:1 metal to ligand stoichiometry (**Zn@DDSQ_Ta**). The appearance of the novel emission band was accompanied by a modification in the color of the solution, which passed from blue to light-cyan as a consequence of the emission contribution centered at 470 nm (see Figure 3).

Compound **DDSQ_Tb** behaves similarly, and the formation of a **Zn@DDSQ_Tb** complex can be claimed as well (see Figure S34 in the Supporting Information). Figure S35 shows the UV–vis spectra of Zn(OTf)_2_.

Once the coordinating properties in the presence of Fe^2+^ and Zn^2+^ cations were evaluated, quantitative experiments in the presence of europium(III) trifluoromethanesulfonate (Eu(OTf)_3_) were performed.

**Figure 4:**
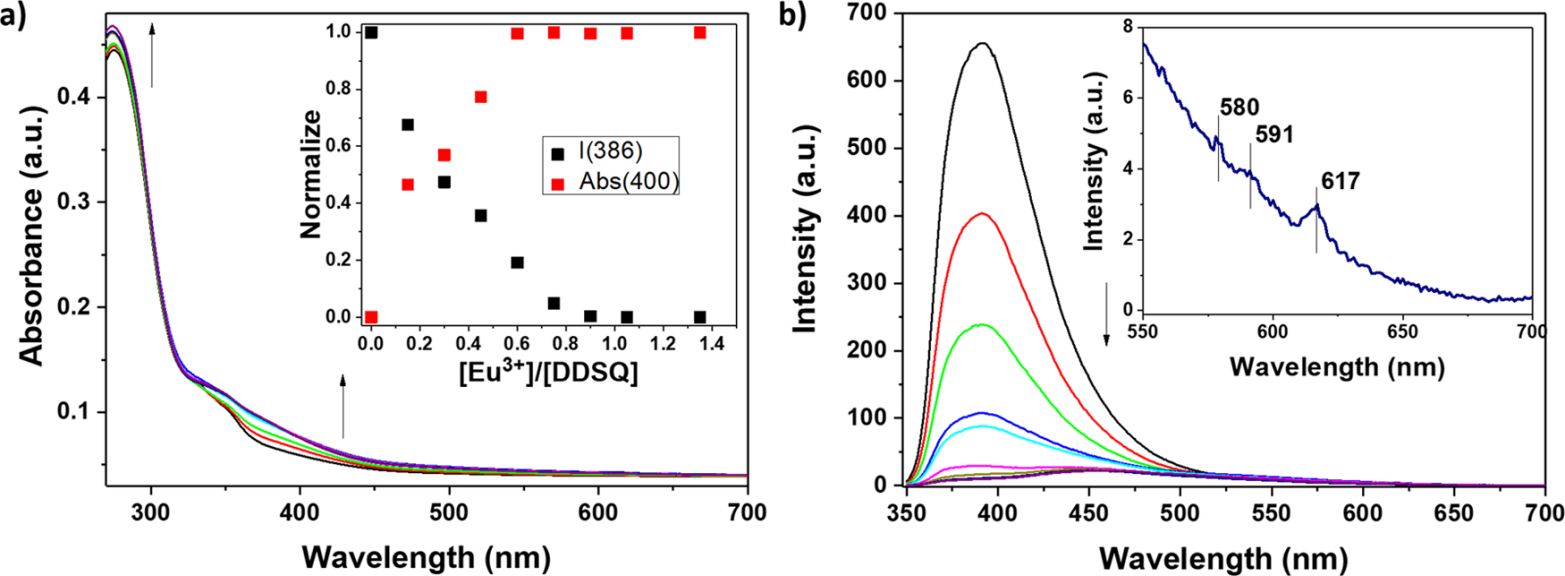
(a) UV–vis absorption spectra of the **DDSQ_Ta** compound in CH_2_Cl_2_ (1 × 10^–6^ M) upon titration with Eu(OTf)_3_ in EtOH (4.17 × 10^–4^ M). The inset shows the normalized absorption changes at 400 nm (red squares) and the normalized emission intensity changes at 386 nm (black squares). (b) Emission spectra of the **DDSQ_Ta** sample in CH_2_Cl_2_ (1 × 10^–6^ M) upon titration with Eu(OTf)_3_ in EtOH (4.17 × 10^–4^ M). The inset shows the emission spectra in the range of 550–700 nm. λ_ex_ = 310 nm, OD = 0.14, and slits = 5 nm.

As seen in Figure 4, on addition of an increasing amount of Eu^3+^ to a solution of ligand **DDSQ_Ta**, the variation of the two absorption bands at 290 and 330 nm in the UV–visible region was immediately visible. As described previously, these two bands are associated with the typical π–π* transition of terpyridine and to the formation of the metal/ligand complex, respectively.

In the inset of Figure 4, the plot of the normalized variation of the absorption and emission bands is presented. As anticipated in the Introduction, europium cations may accommodate up to nine coordination sites (corresponding to three terpyridine molecules) in their coordination shell. In this case, a plateau in correspondence of 0.66 equiv of Eu^3+^ per DDSQ ligand is expected. However, as can be clearly seen (Figure 4a), a plateau is reached at c.a. 0.8 equiv of the metal cation, suggesting that the equilibrium between different species (most probably **3Eu@3DDSQ_Ta**, **2Eu@3DDSQ_Ta**, and **Eu@3DDSQ_Ta**) could be present in the solution. In the case of a lower Eu/ligand ratio, the coordination shell of europium is most probably completed by solvent molecules. Similar behavior was already reported in the literature.^75,76^ Interestingly, in the emission spectra together with the progressive decrease of the contribution centered at 380 nm, the characteristic Eu^3+^ line like emission in the region between 580 and 700 nm was observed (Figure 4b). It should be noted that in previous experiments in the presence of monofunctionalized silsesquioxanes, the typical lanthanide emission related to the f–f electronic transition was not visible.^31^ This behavior suggests that the **DDSQ_Ta** ligands display stronger coordination properties. As a consequence of the appearance of the typical line-like emission, a modification of the emission color from blue (typical of the free **DDSQ_Ta** ligand) to light-yellow (Eu-complexed **DDSQ_Ta**) was observed.

Previously reported^75,76^ polymeric structures or as a function of the amount of the metal cation involved in the coordination, terpyridine-based lanthanide complexes may display green-yellowish or yellow emission, which in some case can appear white to the eyes.

As expected, similar results were obtained with the **DDSQ_Tb** sample (see Figure S36 in the Supporting Information). To better understand the formation of the novel complexes of Eu^3+^ with **DDSQ_Ta** and **DDSQ_Tb**, a novel set of experiments employing a mixture of solvents of different polarities (CH_3_CN (97%):CH_2_Cl_2_ (3%)) were performed as well. Figure S37 shows the UV–vis spectra of Eu(OTf)_3_.

Figure S38 (in the Supporting Information) shows a nonresolved emission centered at 617 nm clearly visible in the novel mixture of solvents, thus confirming the role played by the solvent on the emission of DDSQ structures. This result is in agreement with previous studies involving POSS-functionalized nanostructures.^31^

**Figure 5:**
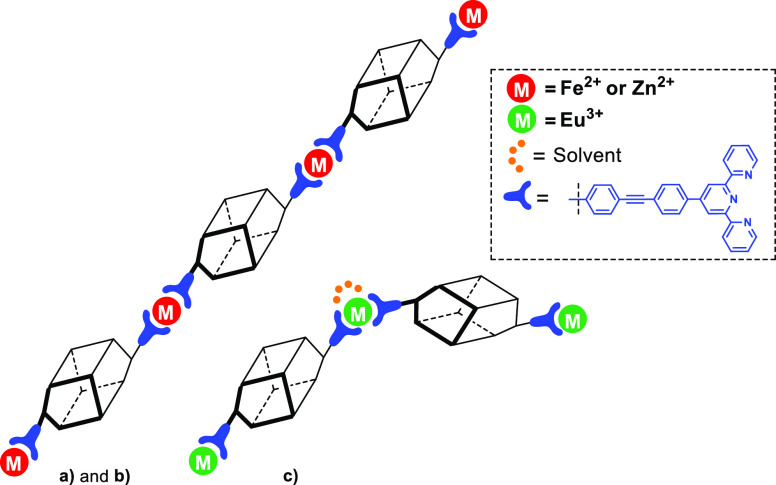
Schematic representation of the complexes (a) **Fe@DDSQ_Tb**, (b) **Zn@DDSQ_Tb**, and (c) **2Eu@3DDSQ_Tb**.

A possible schematic representation of the different complexes is given in Figure 5.

**Figure 6:**
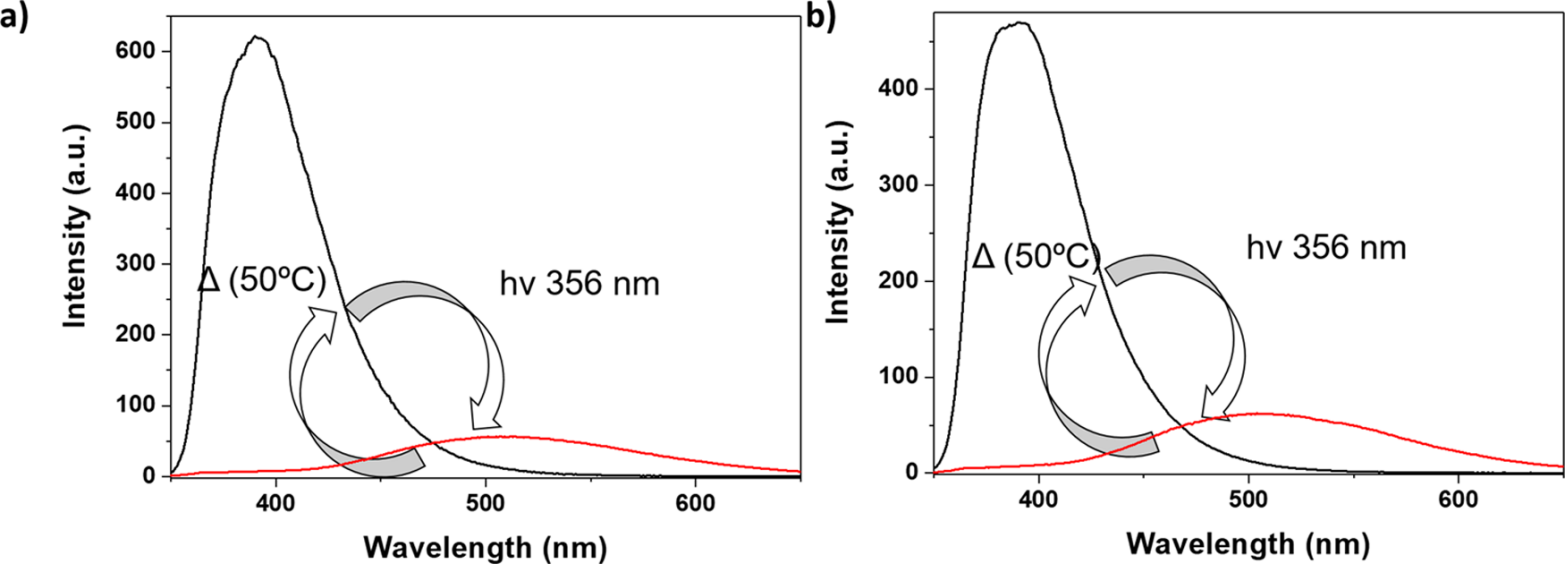
(a) Emission spectra of *E* (black line) to *Z* (red line) reversible isomerization of the **DDSQ_Ta** sample. λ_ex_ = 310 nm and slits = 5 nm. (b) Emission spectra of *E* (black line) to *Z* (red line) reversible isomerization of the **DDSQ_Tb** sample. λ_ex_ = 310 nm and slits = 5 nm.

**Scheme 2:**
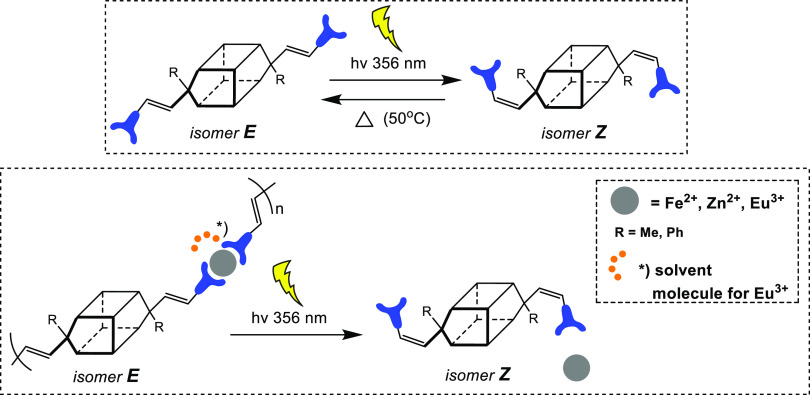
Schematic Representation of the *E* to *Z* and *Z* to *E* Isomerization of the Vinylene Group of **TM@DDSQ_Ta-b**

Moreover, both novel DDSQs present an *E* carbon–carbon double bond that can be isomerized to the *Z* form (Scheme 2), as previously reported for mono- and octafunctionalized silsesquioxanes.^31,48^ It is known that UV light irradiation may promote *E* to *Z* isomerization of the vinyl group. The *E* to *Z* isomerization was monitored via UV–visible absorption and emission investigation of the samples with terpyridine moieties as it can be seen in Figure 6 and Figures S39 and S40 in the Supporting Information. These results were obtained by irradiating the ***E*-DDSQ_Ta-b** at 356 nm for 1 h. As it can be seen in the fluorescence spectra, after irradiation, the bands centered at 380 and 386 nm disappeared, and in both cases, a new band centered at c.a. 500 nm appears, with a consequent variation of the emitted color, which passes to light-green. In agreement with the literature, this band can be attributed to the *Z* form.^31^

Moreover, we also demonstrate that a reversible *Z* to *E* isomerization can be obtained by heating the solution of ***Z*-DDSQ_Ta-b** at 50 °C overnight (Scheme 2). As seen, after thermal treatment, the bands centered at 500 nm disappear, and the band corresponding to the *E*-DDSQ materials is visible (Figure 6). This process can be repeated several times without detrimental effects on the structure of the ligands. As it can be seen in Figure 6, the intensity of the fluorescence band centered at c.a. 500 nm in ***Z*-DDSQ_Ta-b** is broader than the emission band associated to the *E*-form.^31^

**Figure 7:**
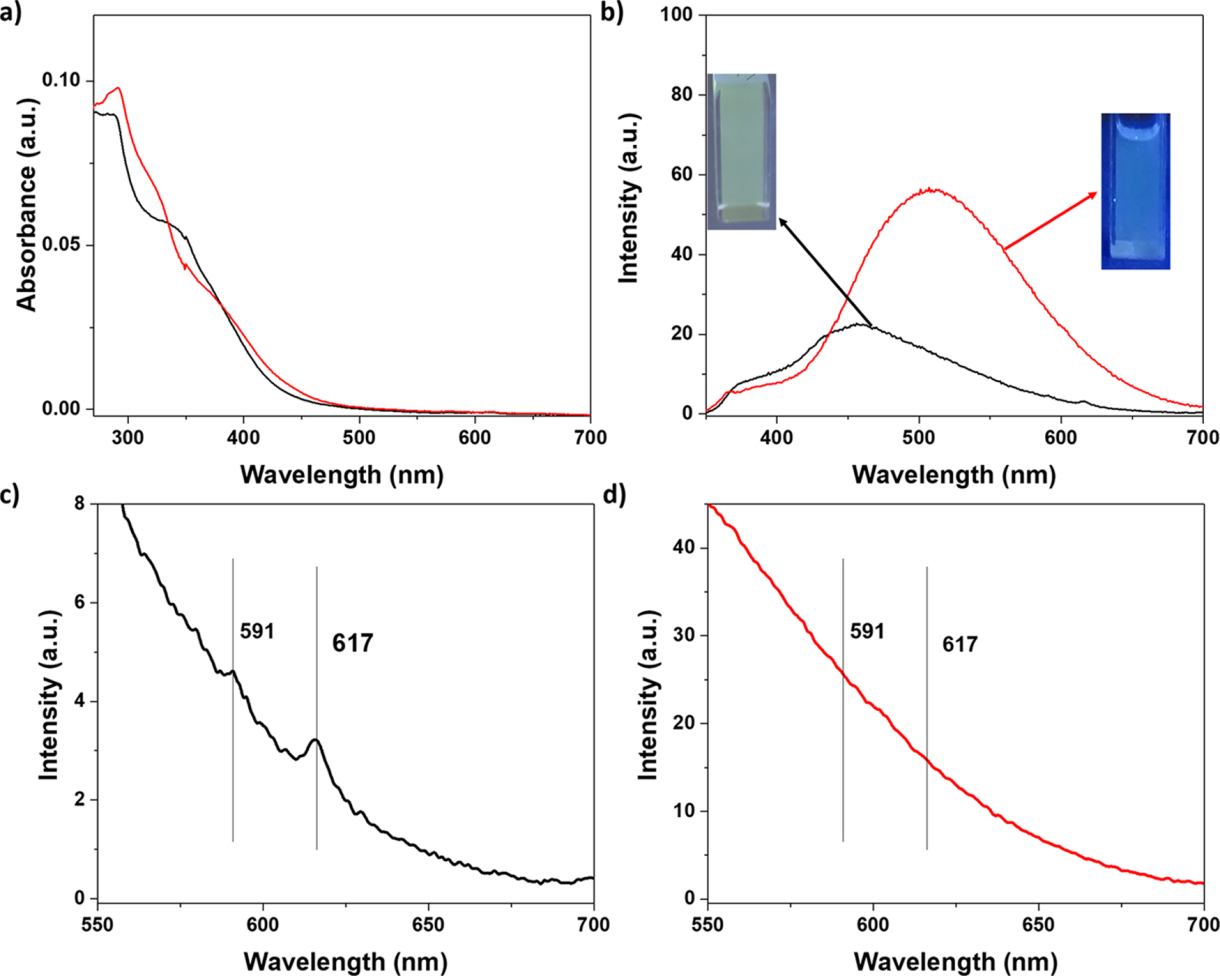
(a) Absorption spectra and (b–d) emission spectra of **Eu@DDSQ_Ta***E* (black line) and *Z* (red line) isomers. λ_ex_ = 310 nm and slits = 5 nm.

The *E* to *Z* isomerization was performed also by employing the **DDSQ_Ta-b** ligands complexed with Eu^3+^ (Figure 7 and Figure S41). As seen in the figures, the typical emission band corresponding to the **Eu@*E*-DDSQ_Ta-b** disappeared, and the emission band related to the ***Z*-DDSQ_Ta-b** isomer is clearly observed in both cases. The signals corresponding to the Eu^3+^ emission disappear, indicating that probably, the lanthanide cations are at least partially released in solution. The final color of the solution corresponds to the one observed for the uncomplexed **DDSQ_Ta** ligand in its *Z* form. This observation constitutes further proof of a possible partial release of the Eu^3+^ ions in solution. Analogous experiments were performed by employing the **Zn** and **Fe@*E*-DDSQ_Ta-b** (Figures S42–S45). In both cases after irradiation at 310 nm, an evident modification of the emission spectra was clearly detected with the appearance of the band corresponding to the *Z* isomer.

Interestingly, also in these cases, a slight variation of the emitted color was also observed. This behavior is particular appealing, especially if applications of these systems in the field of photochromism are envisaged.

The DDSQ scaffold confers stability (with respect to the monomer alone) to polymeric fibers (see below) without affecting negatively the photophysical properties. Moreover, it represents also an interesting core providing switchable properties thanks to the presence of the terpyridine-functionalized double bond.

The series of colors obtained from the emission of the **DDSQ_Ta** ligand before and after complexation with the different metal cations is presented inFigure 8.

**Figure 8:**
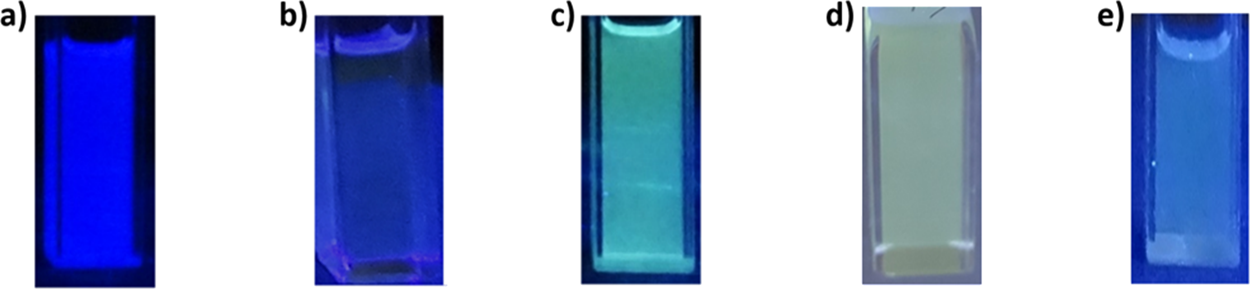
Color displayed for samples (a) ***E*-DDSQ_Ta**, (b) **Fe@DDSQ_Ta**, (c) **Zn@DDSQ_Ta**, (d) **2Eu@3DDSQ_Ta**, and (e) ***Z*-DDSQ_Ta** under UV light emission.

Until now, no major differences between the two DDSQ ligands were evidenced. However, the presence of hindered phenyl moieties in the **DDSQ_Tb** ligand could result in a more dispersed polymeric network after complexation.

### Transmission and Scanning Electron Microscope (TEM and SEM) Investigation

**Figure 9:**
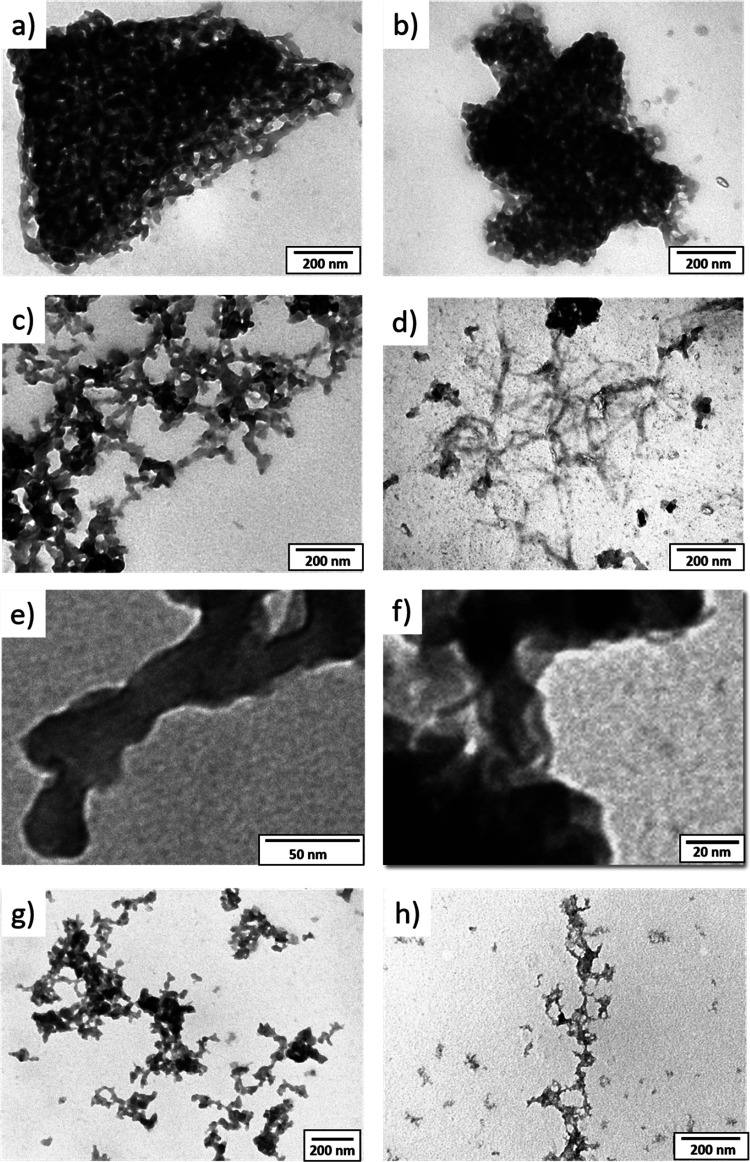
Transmission electron microscopy images of lyophilized (a) **DDSQ_Ta**, (b) **DDSQ_Tb**, (c,e) **Fe@DDSQ_Ta**, (d,f) **Fe@DDSQ_Tb**, (g) **Fe@DDSQ_Ta**, and (h) **Fe@DDSQ_Tb** after sonication.

To shed light on the possible formation of the polymeric nanofibers, transmission electron microscopy (TEM) investigation was performed. As seen in Figure 9, before complexation, both **DDSQ_Ta-b** ligands are randomly organized in compact aggregates (Figure 9a,b). After complexation with iron (Figure 9c,d), some organizations typical of polymeric structures can be observed for all samples. Careful examination at higher magnification allows highlighting that the polymeric aggregates are the result of a combination of entangled 1D nanofibers (Figure 9e,f). The difficulty related to the electron microscopy investigation is due to the low contrast between the mainly organic nanofibers and the background (constituted by an organic polymeric film covering the TEM grids) as well as to the low stability of the fibers under the electron beam at high magnification. As expected, the **DDSQ_Tb** ligands produce more dispersed nanostructures. This behavior was even more evident after sonication of the solution before deposition on the TEM grid (Figure 9g,f). In the case of ligand **DDSQ_Ta**, no significant difference (before and after sonication) was observed, while in the presence of **DDSQ_Tb**, some isolated 1D organization can be identified after sonication. Additional TEM images can be found in the Supporting Information (Figure S46) along with the SEM images for selected samples (Figure S47). However, due to the lower resolution of the SEM analysis as well as the intrinsic limitation of this technique, no better resolution of the entangled nanotubular structure was achieved.

## Conclusions

In summary, an efficient and selective synthetic approach for novel difunctionalized DDSQs with two (styrylethynylphenyl)terpyridine moieties obtained via consecutive silylative and Sonogashira coupling reaction was explored. This is the first example demonstrating the catalytic reactivity of disubstituted DDSQs in Sonogashira coupling, which is an important aspect in revealing the potential application of these silsesquioxanes. Obtained moieties were thoroughly characterized with spectroscopic (NMR and FT-IR), spectrometric (MALDI-TOF-MS), and also XRD (**DDSQb**) analyses. Thermal properties of (ethynylphenyl)terpyridine (**T**) and all synthesized DDSQ derivatives were also verified. Moreover, the correlation between the structure and respective thermal stabilities was appropriately described.

Additionally, 1D metal-silsesquioxane cage structures were obtained via the assembly of di((styrylethynylphenyl)terpyridine)DDSQs playing the role of ligands with three different metal ions (Fe^2+^, Zn^2+^, and Eu^3+^). All the complexes were thoughtfully investigated via UV–vis and fluorescence spectroscopy. The titration experiments revealed that only 1 equiv of the metal is required to completely coordinate the terpyridine DDSQ-based ligands implying the formation of a complex with 1:1 metal to ligand stoichiometry. This behavior along with electron microscopy investigation (TEM) confirmed the formation of self-assembled coordinating 1D polymeric nanofibers. What is more, the presence of styryl groups bridging the silsesquioxane core to the (ethynylphenyl)terpyridine moiety enables reversible *E*–*Z* isomerization of the double bond without detrimental effects on the DDSQ structure, which may be employed to tune the emission properties. This behavior makes DDSQ-based complexes promising candidates for applications in materials chemistry.

## Supporting Information Available

Synthetic procedures, table of isolated compounds, analytical data of obtained compounds (^1^H, ^13^C, and ^29^Si NMR spectra (Figures S1–S15), IR MALDI, and EA), results and figures of thermal analysis (TGA, DTG, and TGA/DSC (Figures S16–S29)), absorption–emission analysis (Figures S30–S45), TEM images (Figure S46), SEM images (Figure S47), and CCDC 2048724 (PDF)
