# Supplementary material for: Double-Decker Silsesquioxanes Self-Assembled in One-Dimensional Coordination Polymeric Nanofibers with Emission Properties
Source: ACS Appl Mater Interfaces. 2021 May 7;13(19):22806–18. doi: 10.1021/acsami.1c02510 (PMC8289186; doi:10.1021/acsami.1c02510)
Supplement: Supplementary file 1 — am1c02510_si_001.pdf [file am1c02510_si_001.pdf]

Supporting Information

Double-Decker Silsesquioxanes Self-Assembled in One-Dimensional Coordination Polymeric Nanofibers with Emission Properties

Julia Duszczak,‡[a] Katarzyna Mituła, ‡[a] Andrea Santiago-Portillo,*[b] Loraine Soumoy,[b] Monika Rzonsowska,[a] Rafał Januszewski,[c] Luca Fusaro,[b] Carmela Aprile,*[b] and Beata Dudziec*[a]

[a] Department of Organometallic Chemistry, Faculty of Chemistry, Centre for Advanced Technologies, Adam Mickiewicz University in Poznan, Uniwersytetu Poznanskiego 8 and 10, 61-614 Poznan, Poland.
[b] Department of Chemistry, University of Namur, Rue de Bruxelles 61, 5000 Namur, Belgium.
[c] Department of Chemistry and Technology of Silicon Compounds, Faculty of Chemistry, Centre for Advanced Technologies, Adam Mickiewicz University in Poznan, Uniwersytetu Poznanskiego 8 and 10, 61-614 Poznan, Poland.

* E-mail: beata.dudziec@gmail.com, carmela.aprile@unamur.be, andrea.santiago@unamur.be

‡Julia Duszczak and Katarzyna Mituła contributed equally.

Table of contents:

1. General Considerations…………………………………………………………….. ••• S - 2-

1.1 Synthetic procedures……………………….…………………………................................ ••• S - 2-

2. A list of isolated compounds………..………………………………………..………… ••• S - 4-

3. Analytical data of obtained compounds………………………….................................. ••• S - 5-

3.1 Thermal analysis of reagents **DDSQa-b** and **DDSQ_Ta-b**……………………….…... ••• S - 16-

4. Results of absorption – emission analysis of **DDSQa-b**, **DDSQ_Ta-b** and **metal@DDSQ-based complexes**………………………..…………………………… ••• S - 21-

5. Results of Transmission and Scanning Electron Microscopy of **DDSQa-b**, **DDSQ_Ta-b** and and **metal@DDSQ-based complexes**……………………………..…. ••• S - 27-

6. References……………………..……………………………………………………... ••• S - 29-

1. General Considerations

1.1 Synthetic procedures

General synthetic procedure for the synthesis of DDSQa-b via silylative coupling reaction

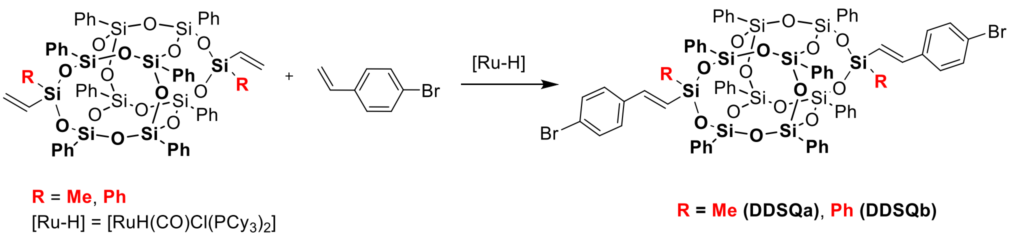

Scheme S1. Synthesis of **DDSQa-b** via silylative coupling reaction.

The respective synthetic protocol is presented for DDSQa and is analogous for DDSQb.
The glass Schlenk reactor equipped with a magnetic stirring bar was evacuated and flushed with argon, a $[RuH(CO)Cl(PCy_3)_2]$ (1.21 mg, 1.662×10^{-6} mmol) and toluene (0.66 mL), then **DDSQ-2(MeSiVi)** (100 mg, 0.0831 mmol) and 4-bromostyrene (0.243 mmol, 35 µL) were added respectively. The reaction was conducted in a closed system for 48 h at 120 °C. After the reaction was completed, the solvent and any excess of volatile reagents were removed under reduced pressure. The crude product (**DDSQa**) was filtered off by column chromatography (silica gel 60, hexane and dichloromethane 1:5) to remove the catalyst, then precipitated in methanol as white solid.

Synthesis of 4'-(4-Ethylnylphenyl)-[2,2':6,2'']terpyridine -T

This compound was synthesized with small changes as previously reported.[1] In this case, 7.68 mmol of NaOH was added to a round-bottom flask with 10 mL of PEG 300 at 0 °C. After that, 7.68 mmol of 2-acetylpyridine was added. After 10 min of magnetic stirred, 3.38 mmol of 4-ethynylbenzaldehyde was added to the solution and it was stirred at 0 ºC for 4 h (instead of reported 2 h). Then, 10 mL of concentrated ammonia aqueous solution was added and the suspension was stirred at room temperature overnight (instead of reported 2 h). Subsequently, the solid was isolated by filtration and washed several times with water and cold ethanol. Extension of reaction time resulted in obtaining better isolation yield (70% instead of reported 44%).

General synthetic procedure for the synthesis of DDSQ_Ta-b via Sonogashira reaction

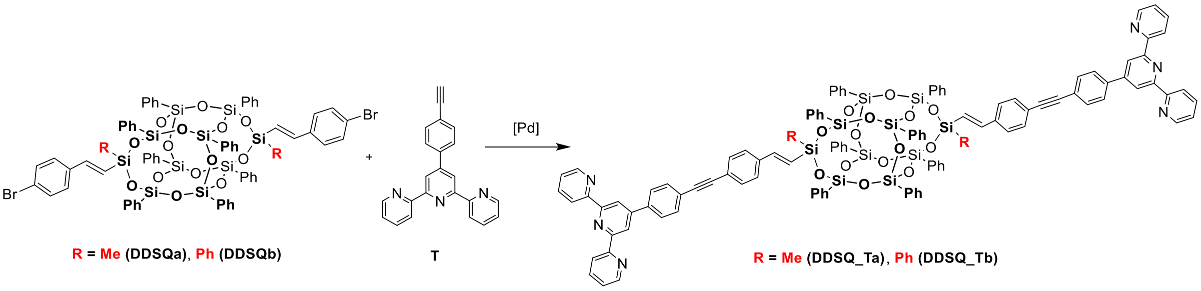

Scheme S2. Synthesis of **DDSQ_Ta-b** via Sonogashira reaction.

The exemplary procedure is presented for the DDSQ_Ta compound and is analogous for DDSQ_Tb.
Into a glass Schlenk reactor equipped with a magnetic stirring bar, **DDSQa** (200 mg, 0.122 mmol), T (122 mg, 0.366 mmol), $Pd(PPh_3)_4$ (51 mg, 0.044 mmol) and CuI (9 mg 0.049 mmol) were added respectively. Then the reactor was flushed with nitrogen for 30 minutes. Afterward dry THF (15 mL) and Et_3N or $(iPr)_2NH$ (5 mL) were added in the presence of nitrogen/argon. The reaction was conducted in a closed system for 24 h at 70 °C. Then the mixture was filtered from insoluble solids and concentrated in vacuo. The crude product (DDSQa) was washed with EtOH and water and then several times (5 x 15 mL) with EtOH using the ultrasound bath for 10 min and centrifuged (10 °C, 4500rpm, 15 min.) receiving brown solid.

General synthetic protocols of preparation of metal@DDSQ-based complexes

The exemplary procedure is presented for **Fe@DDSQ_Ta**.
DDSQ_Ta (3 mg) and 10 mL of CH_2Cl_2 were placed into a glass vial and stirred vigorously using the ultrasound bath for 10 min (1.485×10^{-4} M solution). A solution of Fe(OTf)$_2$ (3 mg, 85% purity) and 20 mL of EtOH was prepared in another vial, also using an ultrasound bath for 10 min (3.63×10^{-4} M solution). Afterward, 67 μL of prepared **DDSQ_Ta** in CH_2Cl_2 was diluted with 9.783 mL of CH_2Cl_2, to obtain 1×10^{-6} M concentration. **DDSQ_Ta** in CH_2Cl_2 was subjected to titration with 5 μL portions of Fe(OTf)$_2$ in EtOH, with 20 min. of vigorous stirring after each addition. The titration was conducted until the equilibrium was reached.

General synthetic protocols of preparation of solids metal@DDSQ-based complexes

The exemplary procedure is presented for **Fe@DDSQ_Ta**.
DDSQ_Ta (15 mg, 7.42×10^{-3} mmol) and 6 mL of CH_2Cl_2 were placed into a glass vial and stirred vigorously using the ultrasound bath for 10 min. A solution of Fe(OTf)$_2$ (3.1 mg, 7.42×10^{-3} mmol 85% purity) and 4 mL of EtOH was prepared in another vial, also using an ultrasound bath for 10 min. After that, the solution of Fe(OTf)$_2$ was placed drop by drop to the vial that contains DDSQ_Ta. The mixture was left at room temperature until it was almost dry. Afterward, the sample was left in the freezer and then it was dried in a freeze dryer.

2. Table S1. A list of isolated compounds

Structure	Compound Abbrev.	Page:
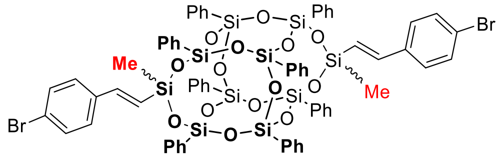	**DDSQa**	**S – 5-6**
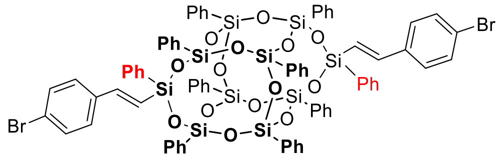	**DDSQb**	**S – 7-9**
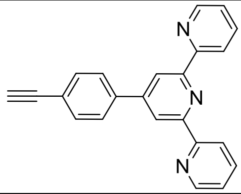	**T**	**S – 10-11**
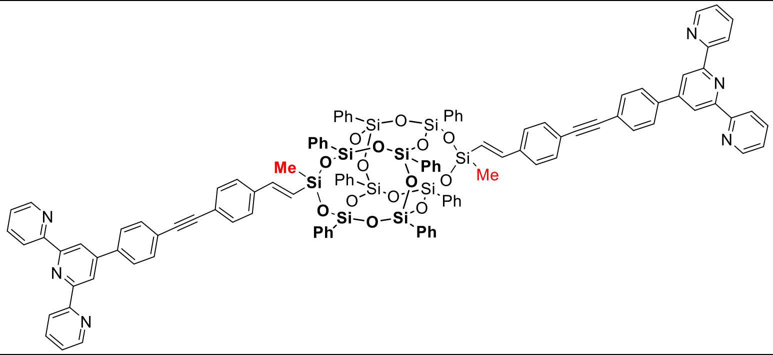	**DDSQ_Ta**	**S – 12-13**
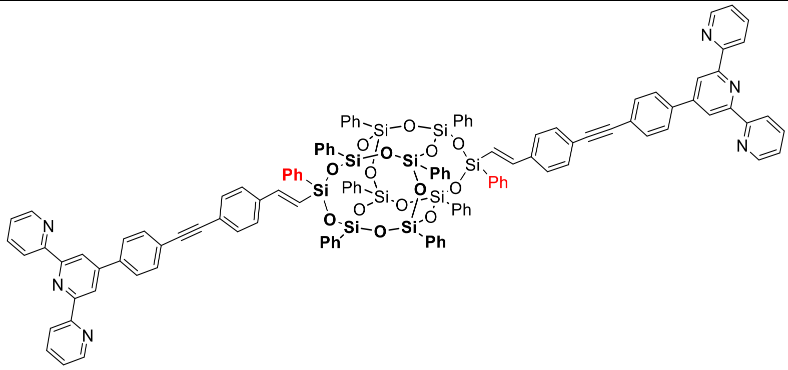	**DDSQ_Tb**	**S – 14-15**

3. Analytical data of obtained compounds

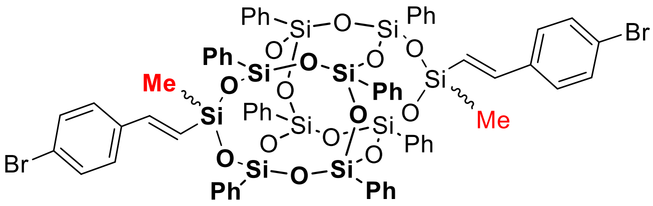

DDSQa White solid. Isolated Yield 82%.

^{1}H NMR (300 MHz, $CDCl_3$, ppm): δ = 0.43 (s, 6H, -Si-CH_3), 6.39 (d, 2H, *J*=19.3Hz, =CH-Si), 6.94-7.46 (m, 42H, C_6H_5-, =CH-C_6H_4-Br), 7.57 (d, 8H, *J*=7.2Hz, C_6H_4-Br).

^{13}C NMR (101 MHz, $CDCl_3$, ppm): δ = -0.66 (-Si-CH_3), 122.45 (*ipso*-C at Br of C6*H*4-Br), 125.14 (=CH-Si), 127.74-128.41, 130.52-131.98, 134.09-134.23, 136.61 (*ipso*-C of C_6H_4-Br), 145.40 (-CH=Ar).

^{29}Si NMR (79 MHz, $CDCl_3$, ppm): δ = -30.47 (-Si-HC=CH-), -78.28, -79.31 (cis), -79.55 (trans), -79.77 (cis) (-Si-C_6H_5).

IR (ATR): v = 3071.77 (m), 3049.73 (m), 3026.31 (m; v_s(C-H phenyl)), 2963.31 (m; v_s(C-H)), 1606.00 (m; v_s(C=C)), 1593.61 (m), 1485.47 (m), 1429.70 (m), 1261.96 (m), 1177.78 (s), 1028.63 (s; v_{as}(Si-O-Si), 997.96 cm^{-1} (s).

MALDI-ToF MS: Calcd. for $C_{66}H_{58}Br_2Na^+O_{14}Si_{10}$: *m/z* 1534.9778 [M + Na^+]. Found: 1534.9791.

Elemental analysis: Found: C, 52.4; H, 3.43.Calc. for $C_{66}H_{58}Br_2O_{14}Si_{10}$: C, 52.3; H, 3.86%

Analytical data correspond with the literature.[2]

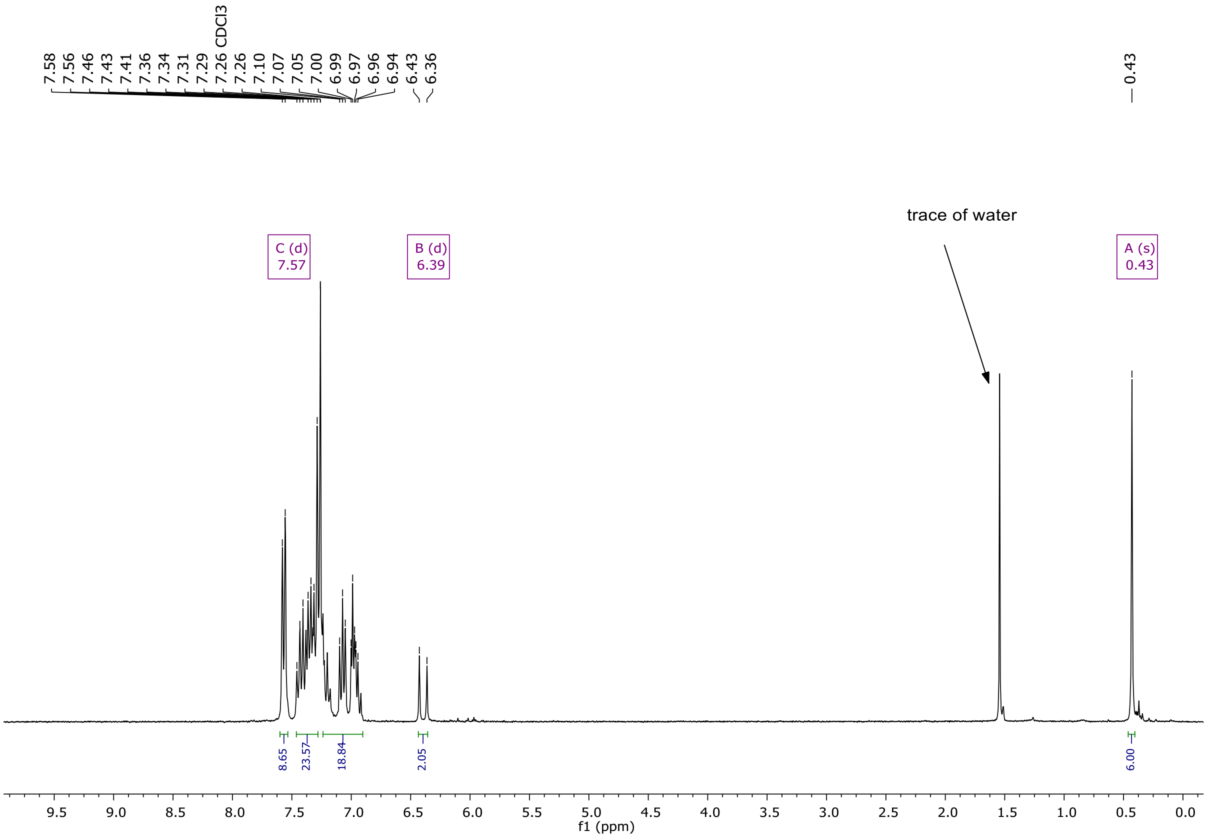

Figure S1. ^{1}H NMR spectra of DDSQa (300 MHz, $CDCl_3$).

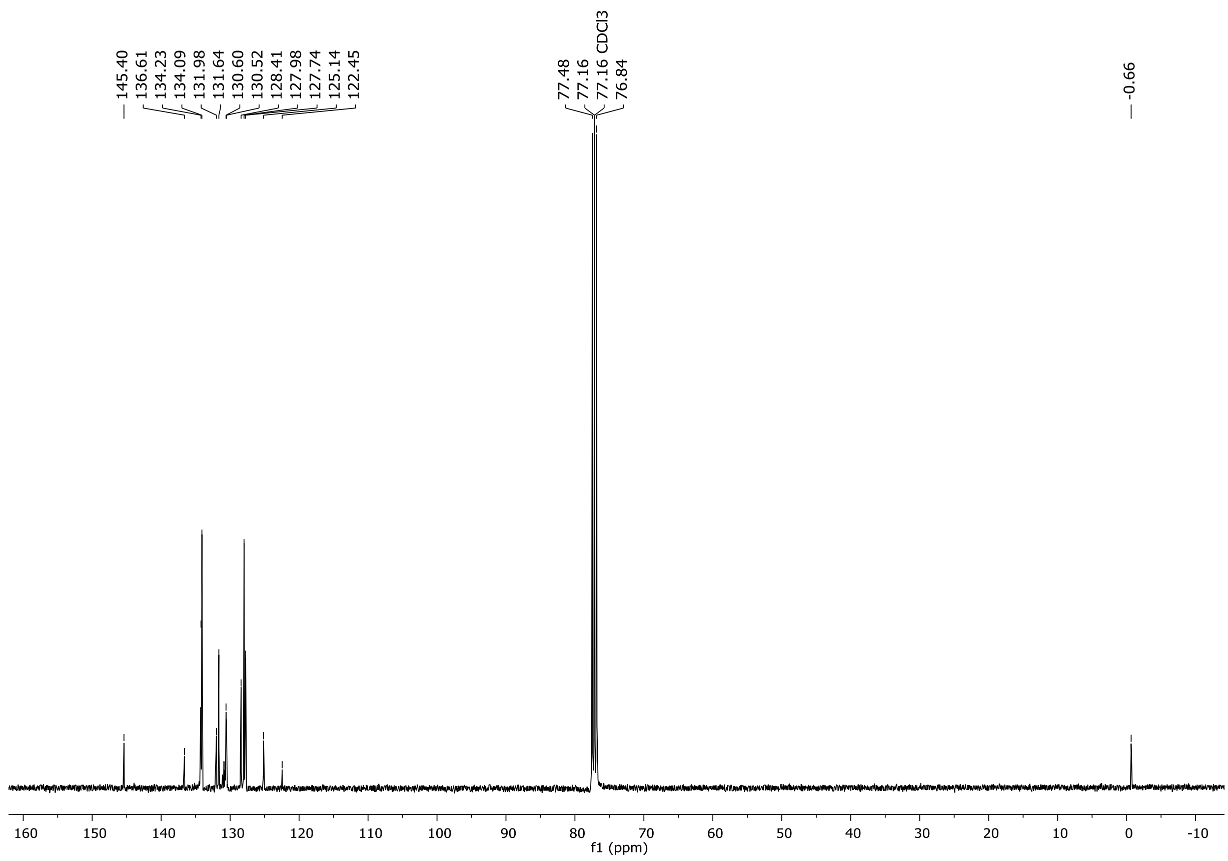

Figure S2. ^{13}C NMR spectra of DDSQa (101 MHz, $CDCl_3$).

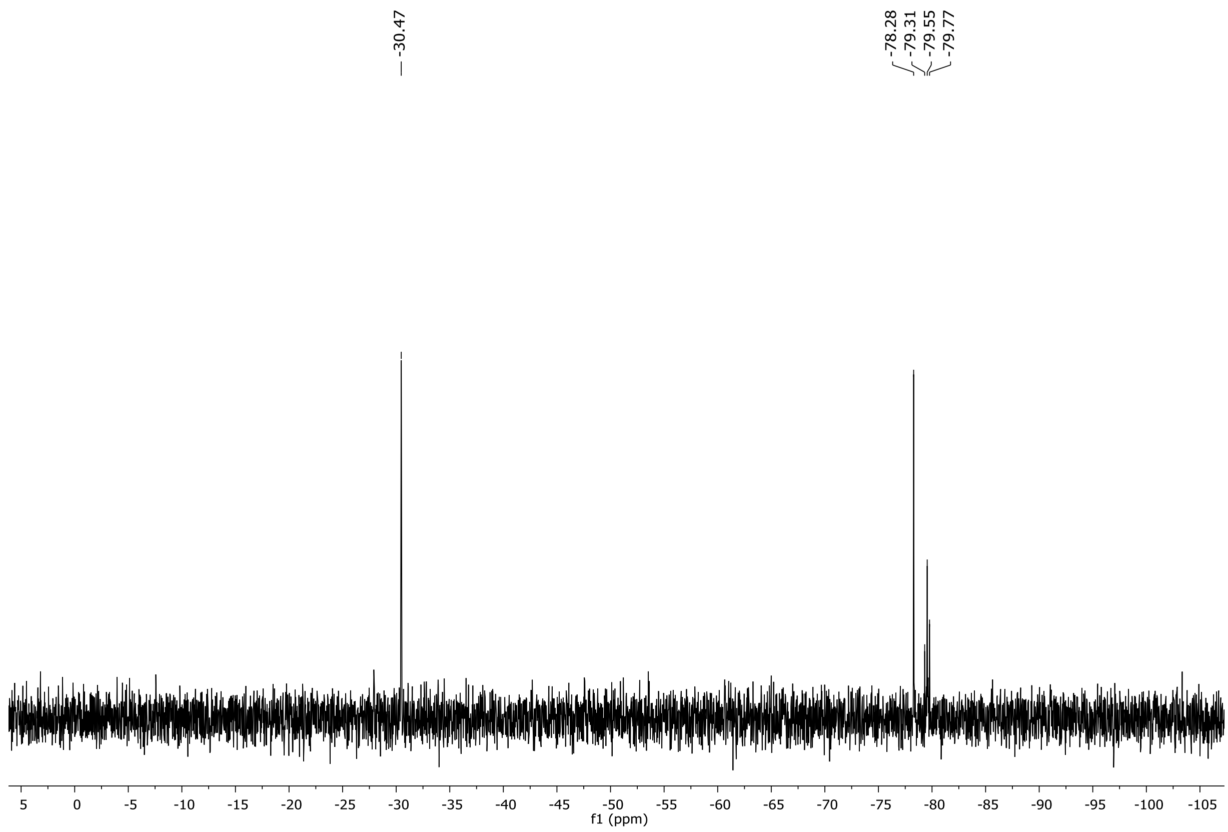

Figure S3. ^{29}Si NMR spectra of DDSQa (79 MHz, $CDCl_3$).

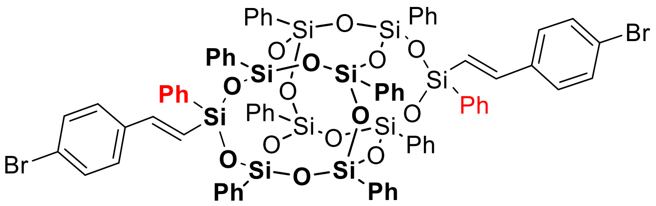

DDSQb White solid. Isolated Yield 90%.

^{1}H NMR (300 MHz, $CDCl_3$, ppm): δ = 6.51 (d, 2H, *J* =19.2Hz, =CH-Si), 6.97-7.70 (m, 60H, C_6H_5- and C_6H_4-Br, =CH-C_6H_4-Br).

^{13}C NMR (101 MHz, $CDCl_3$, ppm): δ = 122.62 (*ipso*-C at Br of C_6H_4-Br), 123.58 (=CH-Si), 127.67-128.52, 130.42-131.71, 134.16-134.24, 136.49 (*ipso*-C of C_6H_4-Br), 146.81 (CH=Ar).

^{29}Si NMR (79 MHz, $CDCl_3$, ppm): δ = -44.83 (-Si-HC=CH-), -77.93, -79.41 (trans) (-Si-C_6H_5).

IR (ATR) *v* = 3071.26 (m), 3045.28 (m), 3005.40 (m; v_s(C-H phenyl)), 1593.49 (w; v_s (C=C)), 1485.34 (m), 1429.22 (m), 1264.56 (m), 1064.69 (s), 1027.21 (s; v_{as} (Si-O-Si), 996.85 cm^{-1} (s).

MALDI-ToF MS: Calcd. for $C_{76}H_{62}$ $Br_2Na^+O_{14}Si_{10}$: *m/z* 1659.0091 [M + Na$^+$]. Found: 1659.0107.

Elemental analysis: Found: C, 55.53; H, 3.66. Calc for $C_{76}H_{62}$ $Br_2O_{14}Si_{10}$: C, 55.66; H, 3.81%

Crystal Data for $C_{76}H_{62}Br_2O_{14}Si_{10}$ (M =1639.97 g/mol): monoclinic, space group P2$_1$/c (no. 14), a = 13.8835(4) Å, b = 13.9833(2) Å, c = 19.2735(5) Å, β = 92.132(2)°, V = 3739.11(15) Å^3, Z = 2, T = 100.01(10) K, μ(MoKα) = 1.309 mm^{-1}, *Dcalc* = 1.457 g/cm^3, 57797 reflections measured (5.826° ≤ 2Θ≤ 57.168°), 8758 unique (R_{int} = 0.0270, R_{sigma} = 0.0211) which were used in all calculations. The final R_1 was 0.0613 (I > 2σ (I)) and wR_2 was 0.1796 (all data).

CCDC **2048724** contains the supplementary crystallographic data for this paper. These data can be obtained free of charge from The Cambridge Crystallographic Data Centre via www.ccdc.cam.ac.uk/data_request/cif.

Analytical data correspond with the literature.[2]

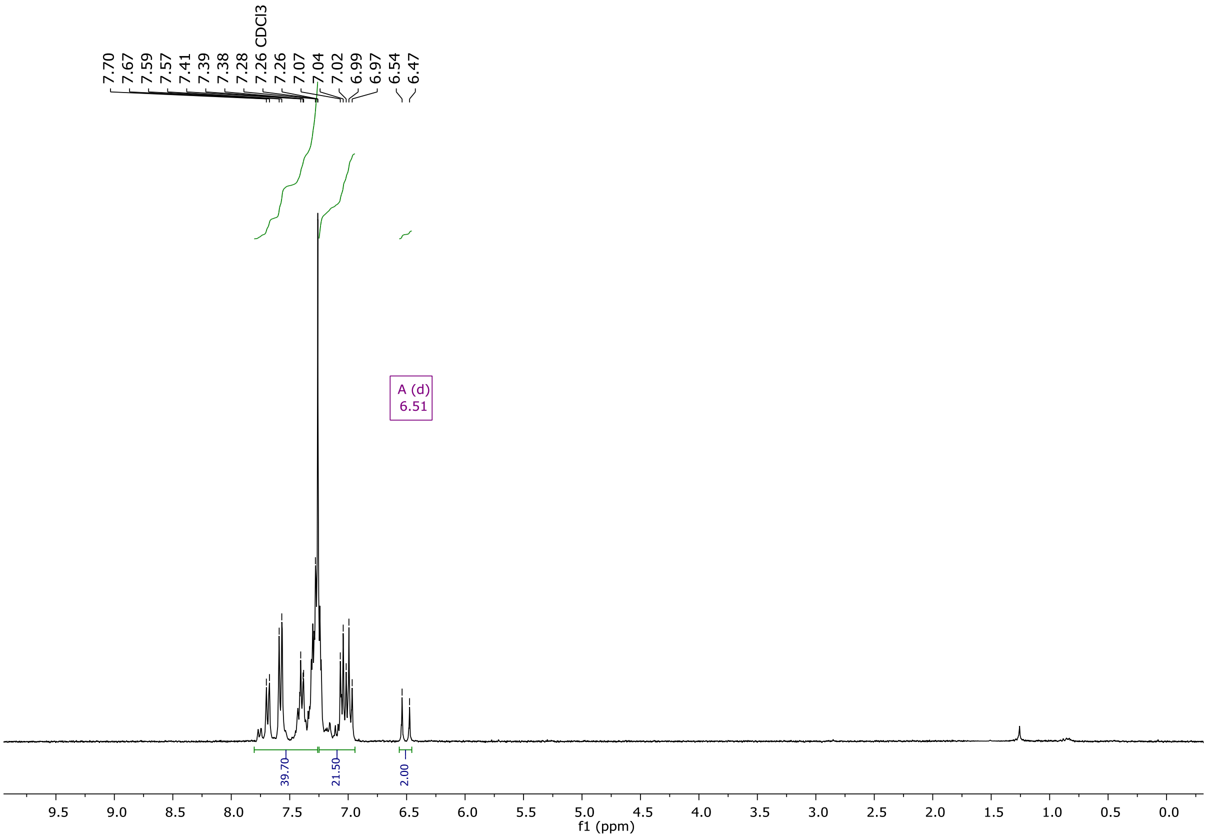

Figure S4. ^{1}H NMR spectra of DDSQb (300 MHz, $CDCl_3$).

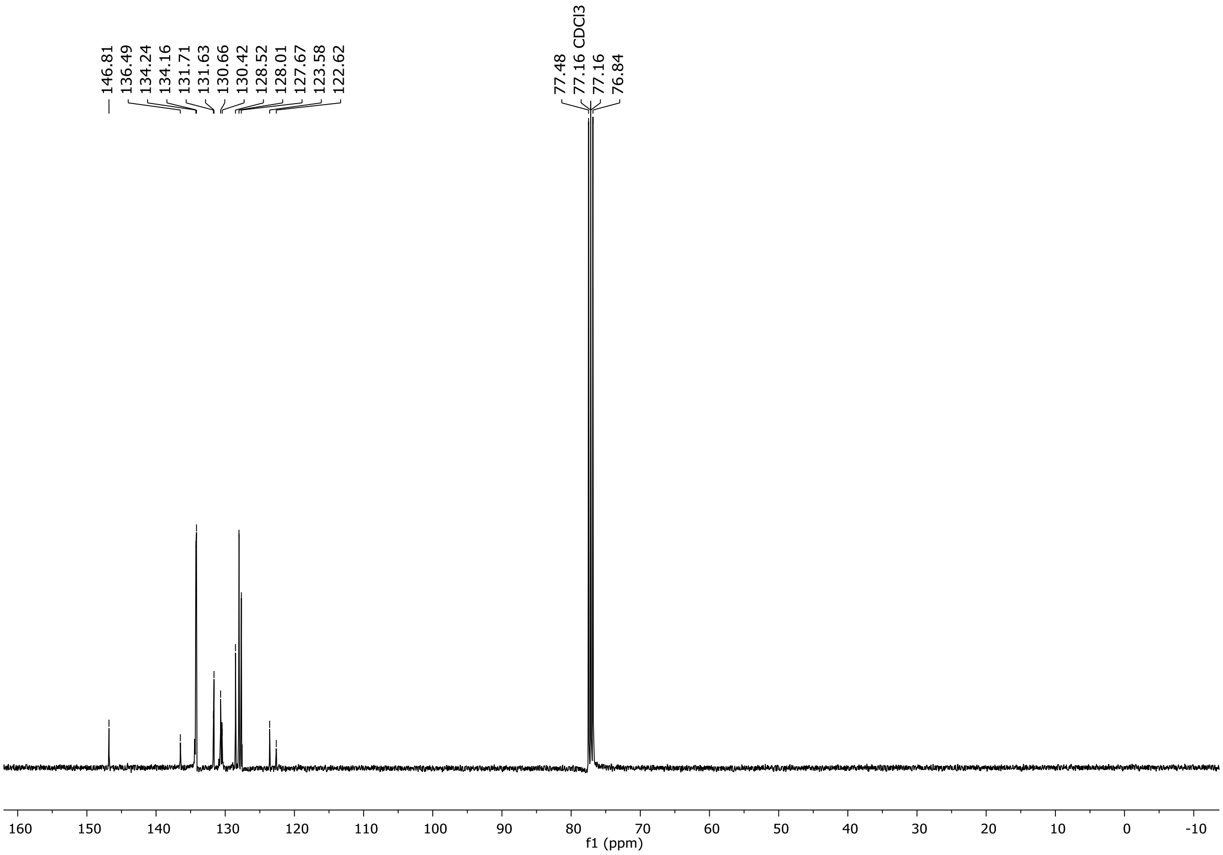

Figure S5. ^{13}C NMR spectra of DDSQb (101 MHz, $CDCl_3$).

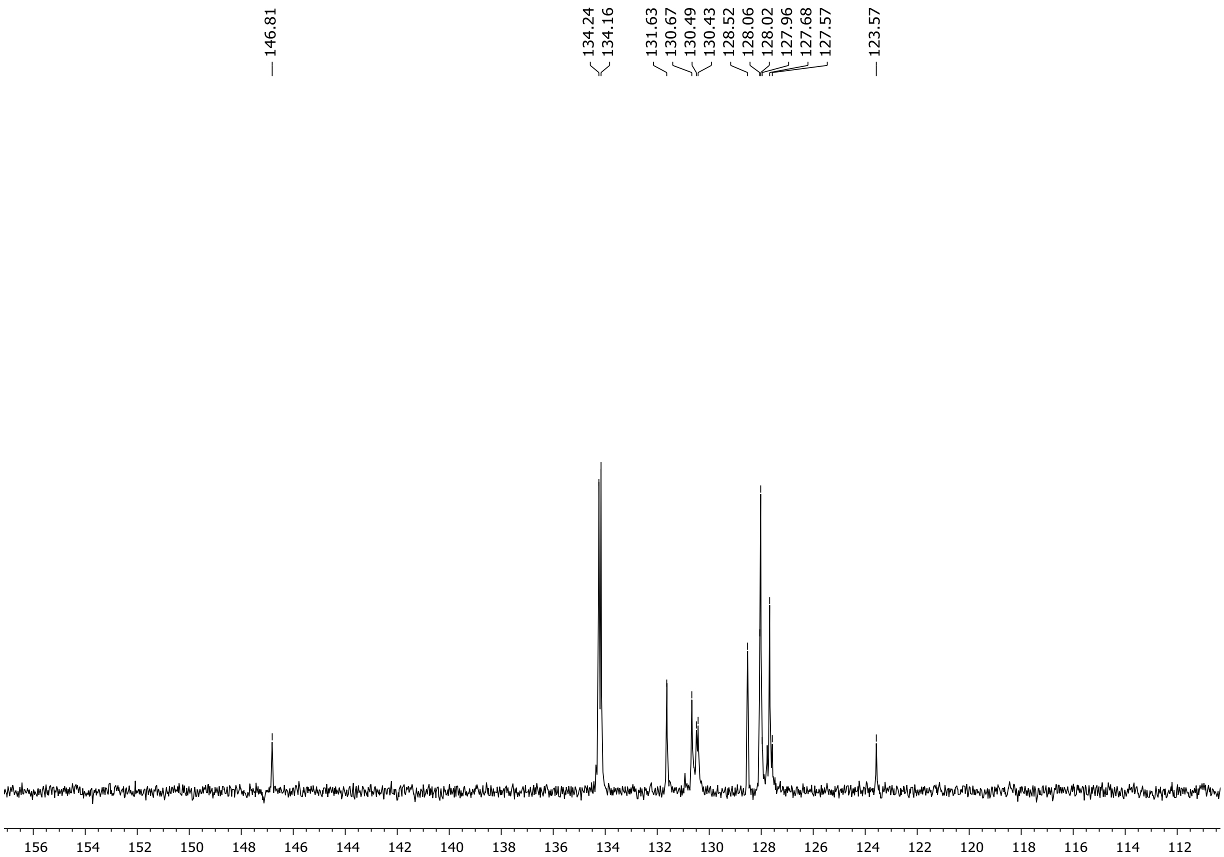

Figure S6. ^{13}C DEPT NMR spectra of DDSQb (76 MHz, $CDCl_3$).

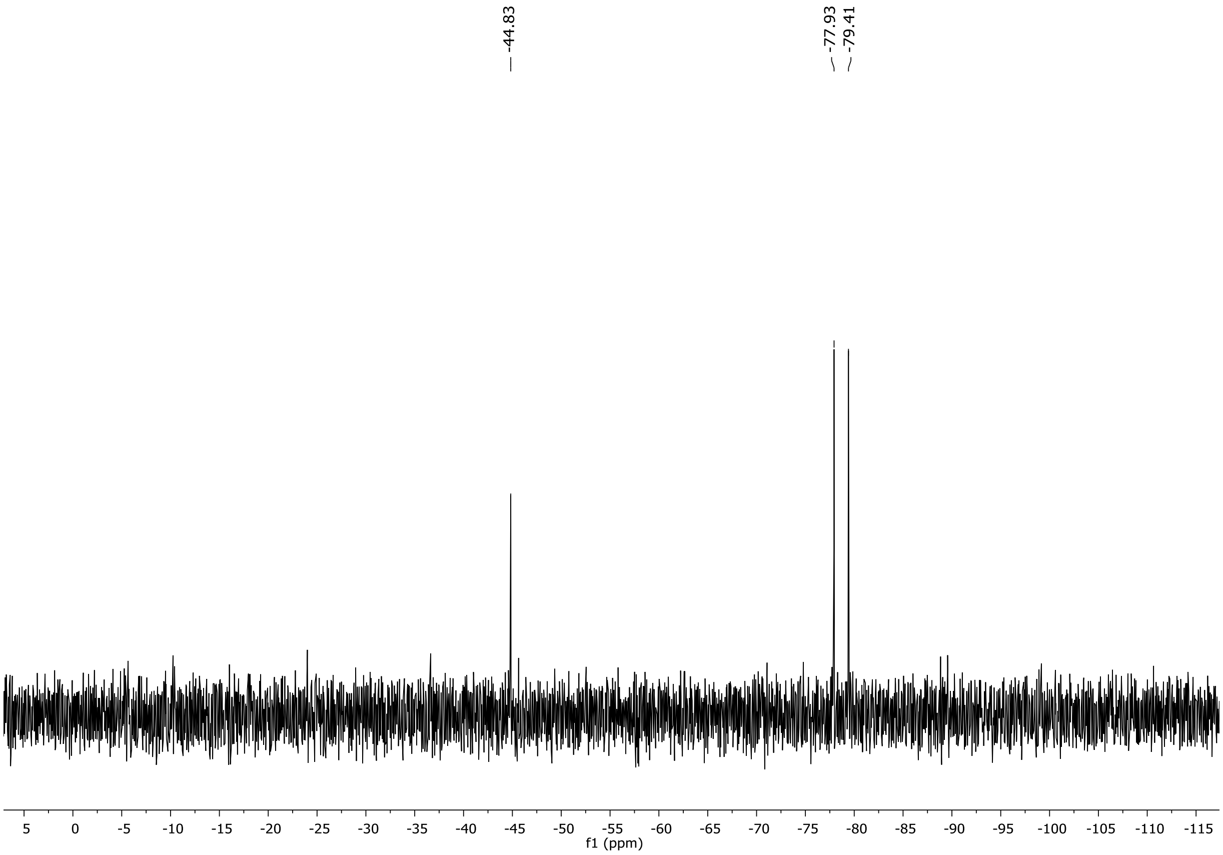

Figure S7. ^{29}Si NMR spectra of DDSQb (79 MHz, $CDCl_3$).

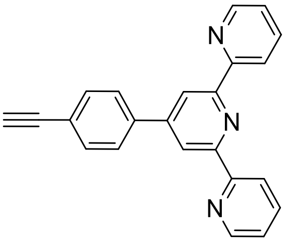

4’-(4-Ethylnylphenyl)-[2,2’:6,2’’]terpyridine, T. Pale yellow solid. Isolated Yield 70%.
^{1}H NMR (300 MHz, $CDCl_3$, ppm): δ = 3.19 (s, 1H, HC≡), 7.34-7.39 (m, 2H), 7.64 (d, 2H, *J* =8.3 Hz), 7.87-7.92 (m, 4H), 8.68 (d, 2H, *J* =7.9 Hz), 8.73 (s, 4H).
^{13}C NMR (101 MHz, $CDCl_3$, ppm): δ = 78.67 (HC≡), 83.40 (≡C-), 118.85, 121.51, 122.94, 124.05, 127.38, 132.83, 137.03, 138.97, 149.29, 149.43, 156.22, 156.23.
Analytical data correspond with the literature.[1]

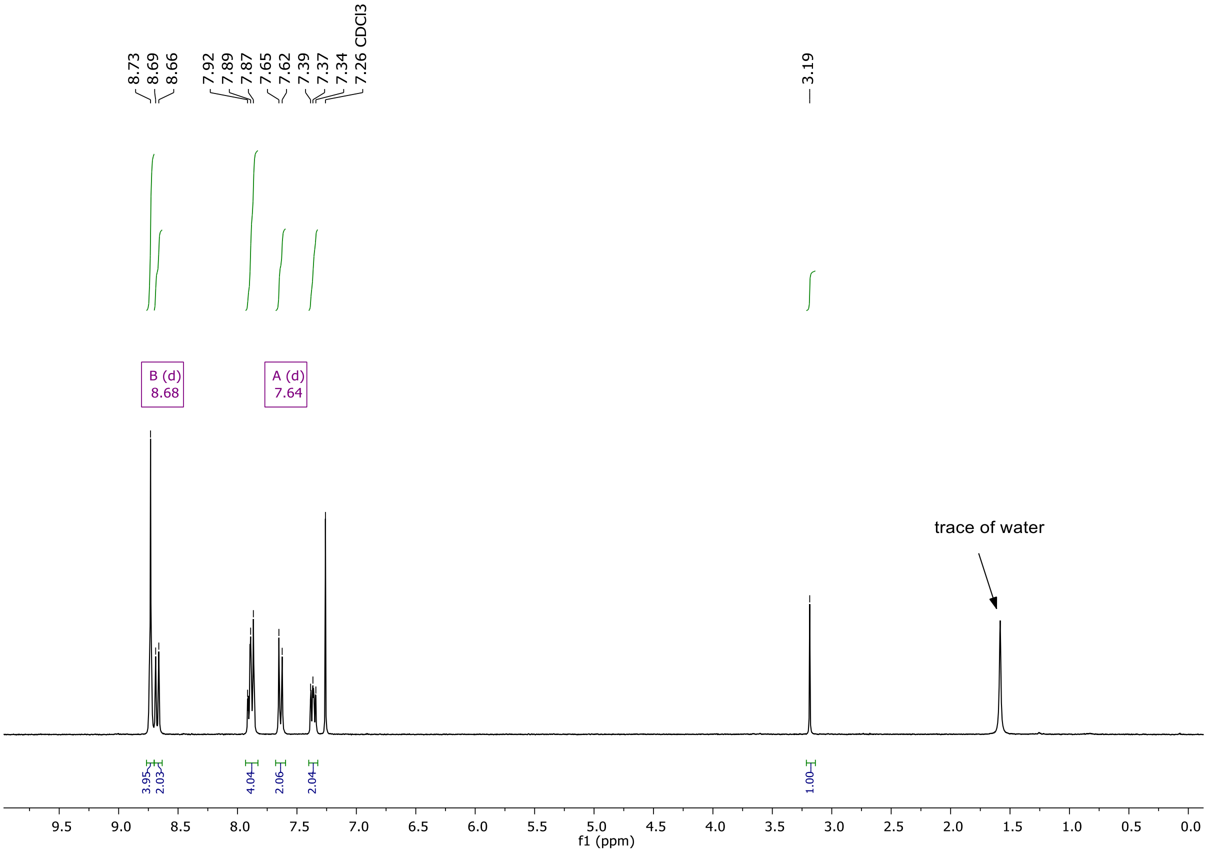

Figure S8. ^{1}H NMR spectra of T (300 MHz, $CDCl_3$).

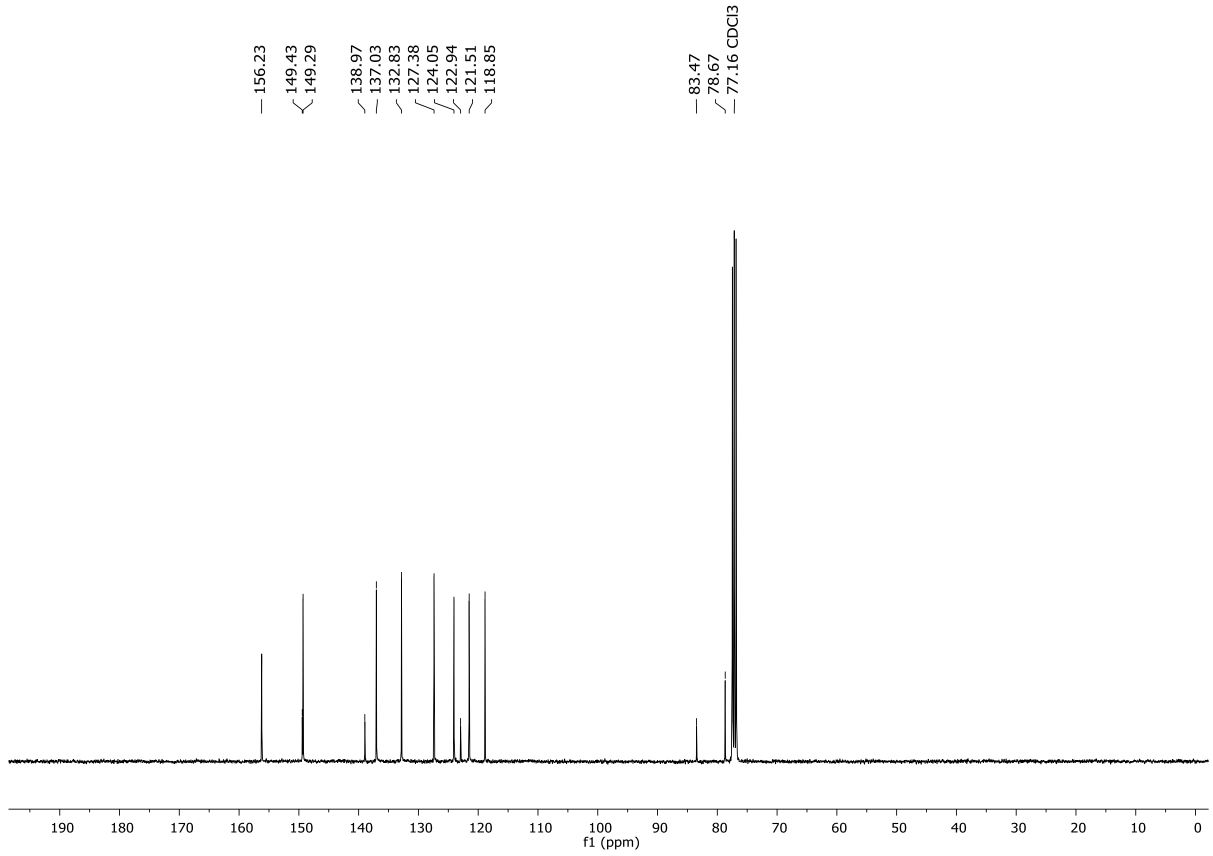

Figure S9. ^{13}C NMR spectra of T (101 MHz, $CDCl_3$).

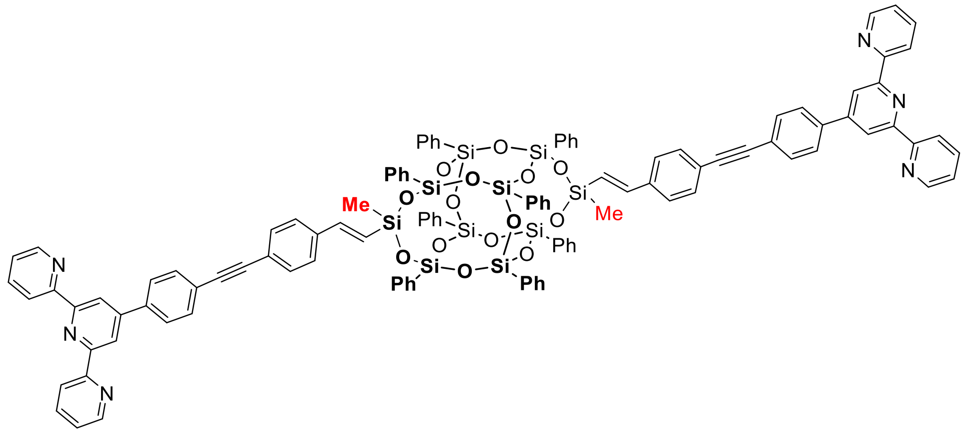

DDSQ_Ta Brown solid. Isolated Yield 68%.

^{1}H NMR (400 MHz, $CDCl_3$, ppm): δ = 0.44 (s, 6H, -Si-CH_3), 6.44-6.49 (m, 2H, =CH-Si), 6.98-8.72 (m, 78H, C_6H_5- and -C_6H_4-, =CH-C_6H_4-, Tpy).

MAS ^{13}C NMR (125 MHz, ppm): δ = -0.56 (-Si-CH_3), 123.2-133.39, 147.52, 155.42 (Ph, Tpy).

MAS ^{29}Si NMR (99.3 MHz, ppm): δ = -35.99 (-Si-HC=CH-), -84.94 (-Si-C_6H_5).

IR (ATR): ν= 3070.45 (m), 3048.67 (m, $ν_s$(C-H phenyl)), 2962.61 (m, $ν_s$ (C-H)), 1601.98 (m; $ν_s$ (C=C)), 1583.32 (m), 1565.60 (m; $ν_s$ (C=N)), 1516.44 (m), 1466.56 (m), 1429.55 (m), 1387.79 (m), 1262.99 (m), 1074.11 (s), 1027.73 (s; $ν_{as}$ (Si-O-Si), 996.98 cm^{-1} (s).

MALDI-ToF MS: Calcd. for $C_{112}H_{86}H^{+}N_6O_{14}Si_{10}$: *m/z* 2019.3967 [M + H^{+}]. Found: 2019.4044.

Elemental analysis: Found: C, 61.79; H, 4.06; N, 3.98. Calc for $C_{112}H_{86}N_6O_{14}Si_{10}$: C, 66.57; H, 4.29; N, 4.16%

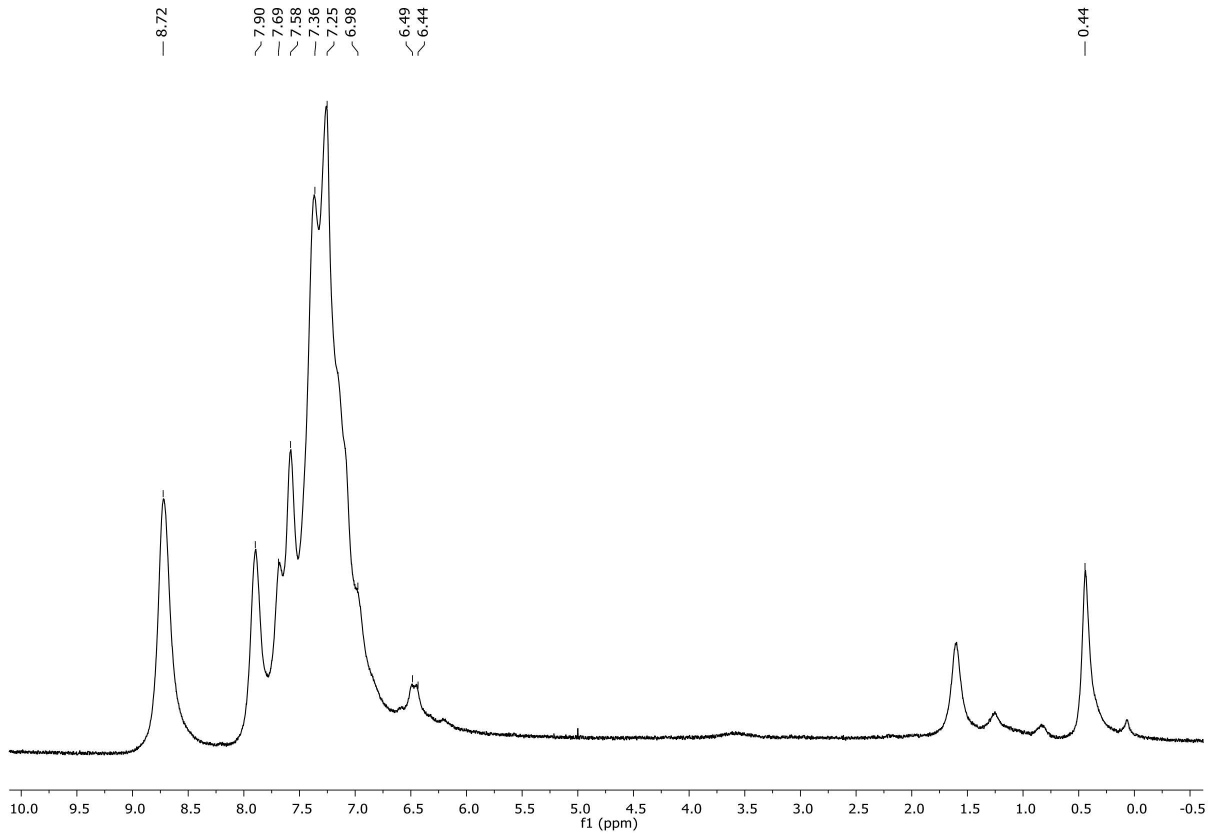

Figure S10. ^{1}H NMR spectra of DDSQ_Ta (400 MHz, $CDCl_3$)

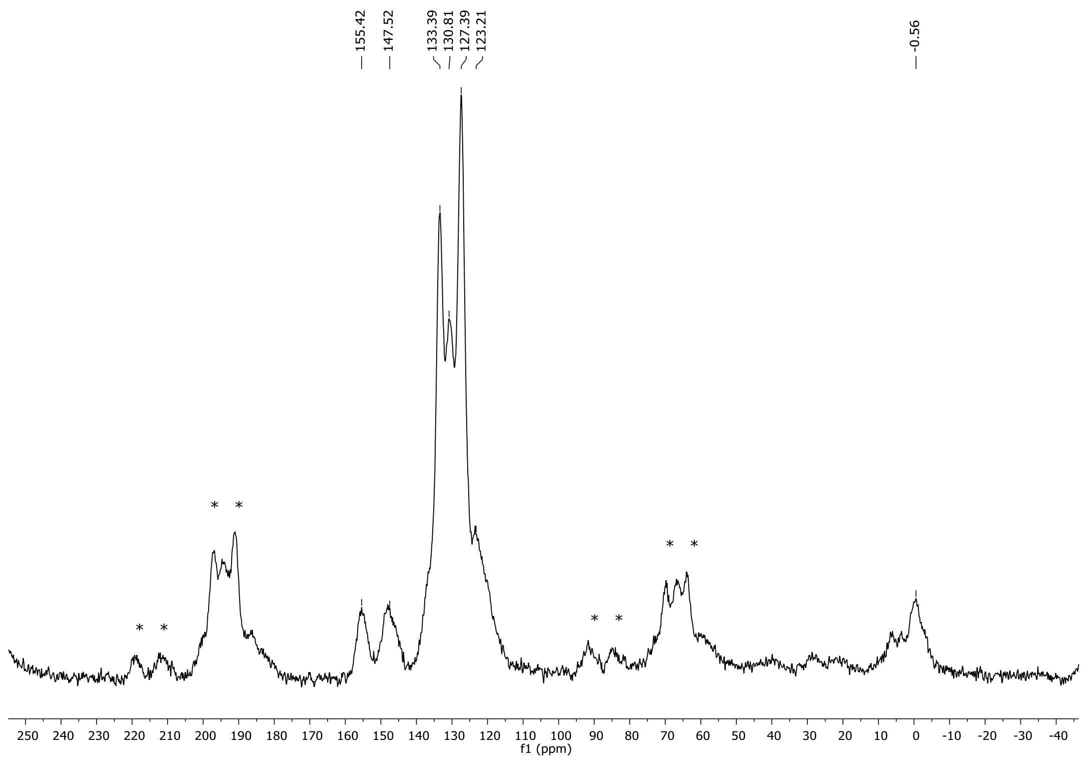

Figure S11. CP-MAS ^{13}C NMR spectra of DDSQ_Ta (125 MHz, 8kHz). Asterisks denote spinning sidebands.

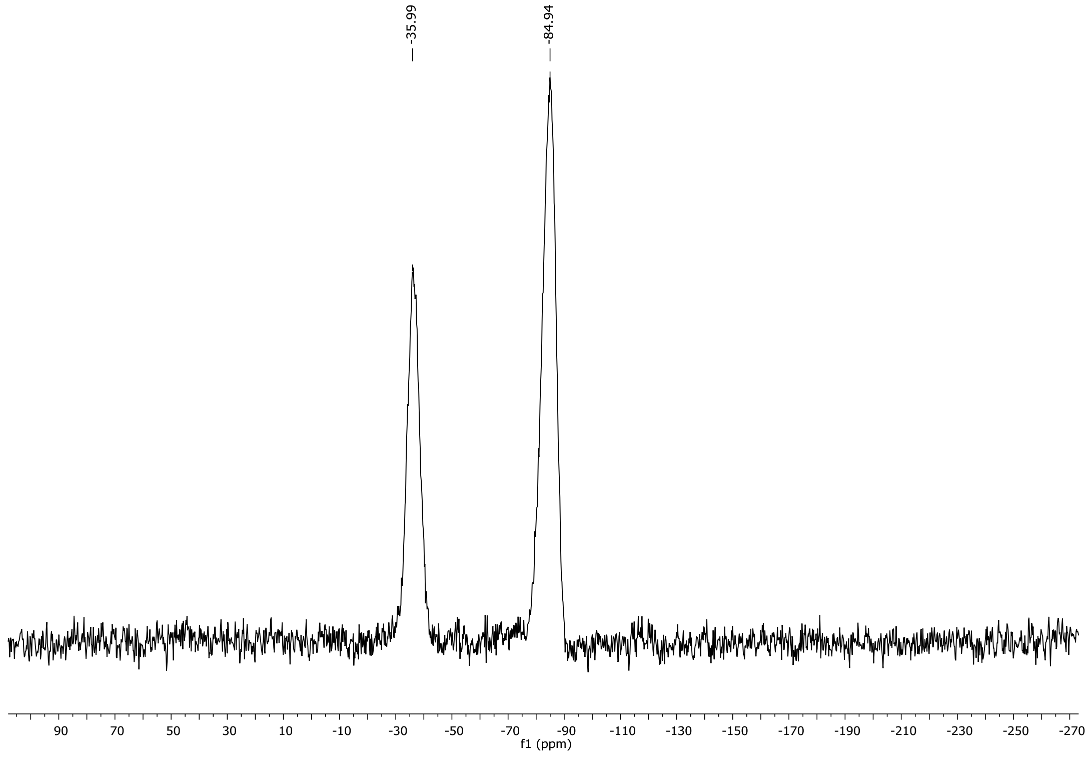

Figure S12. CP-MAS ^{29}Si NMR spectra of DDSQ_Ta (99.3 MHz, 8kHz).

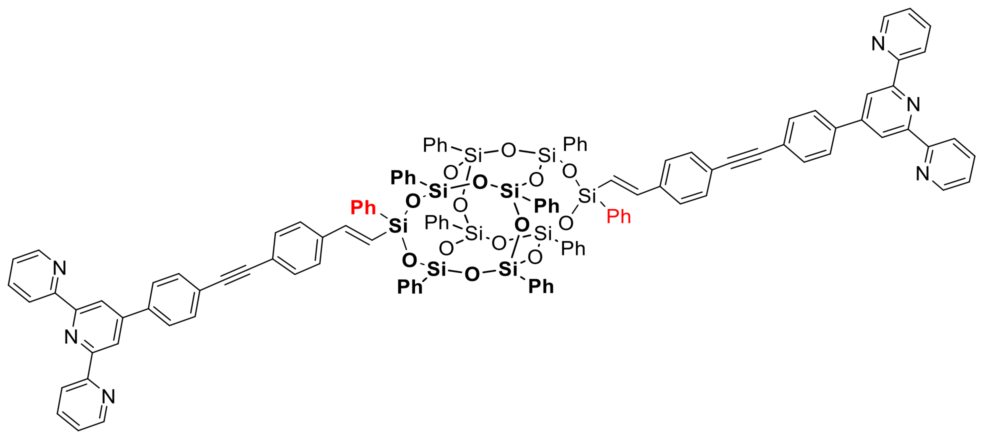

DDSQ_Tb Brown solid. Isolated Yield 72%.

^{1}H NMR (400 MHz, $CDCl_3$, ppm): δ = 6.57-6.62 (m, 2H, =CH-Si), 7.12-8.74 (m, 88H, C_6H_5- and C_6H_4-, =CH-C_6H_4-, Tpy).

MAS ^{13}C NMR (125 MHz, ppm): δ = 112.94-131.13, 145.28, 152.91 (Ph, Tpy).

MAS ^{29}Si NMR (99.3 MHz, ppm): δ = -50.71 (-Si-HC=CH-), -84.21 (-Si-C_6H_5).

IR (ATR): ν = 3070.21 (m), 3048.66 (m, ν_s (C-H phenyl)), 1600.00 (m; ν_s (C=C)), 1583.47 (m), 1564.94 (m; ν_s (C=N)), 1516.30 (m), 1465.80 (m), 1429.20 (m), 1387.49 (m), 1262.99 (m), 1072.80 (s), 1027.17 (s; ν_{as} (Si-O-Si), 996.70 cm^{-1} (s).

MALDI-ToF MS: Calcd. for $C_{122}H_{90}H^{+}N_6O_{14}Si_{10}$: *m/z* 2143.4280 [M + H^{+}]. Found: 2143.4058.

Elemental analysis: Found: C, 67.07; H, 4.04; N, 4.08. Calc for $C_{122}H_{90}N_6O_{14}Si_{10}$: C, 68,32; H, 4.23; N, 3.92%

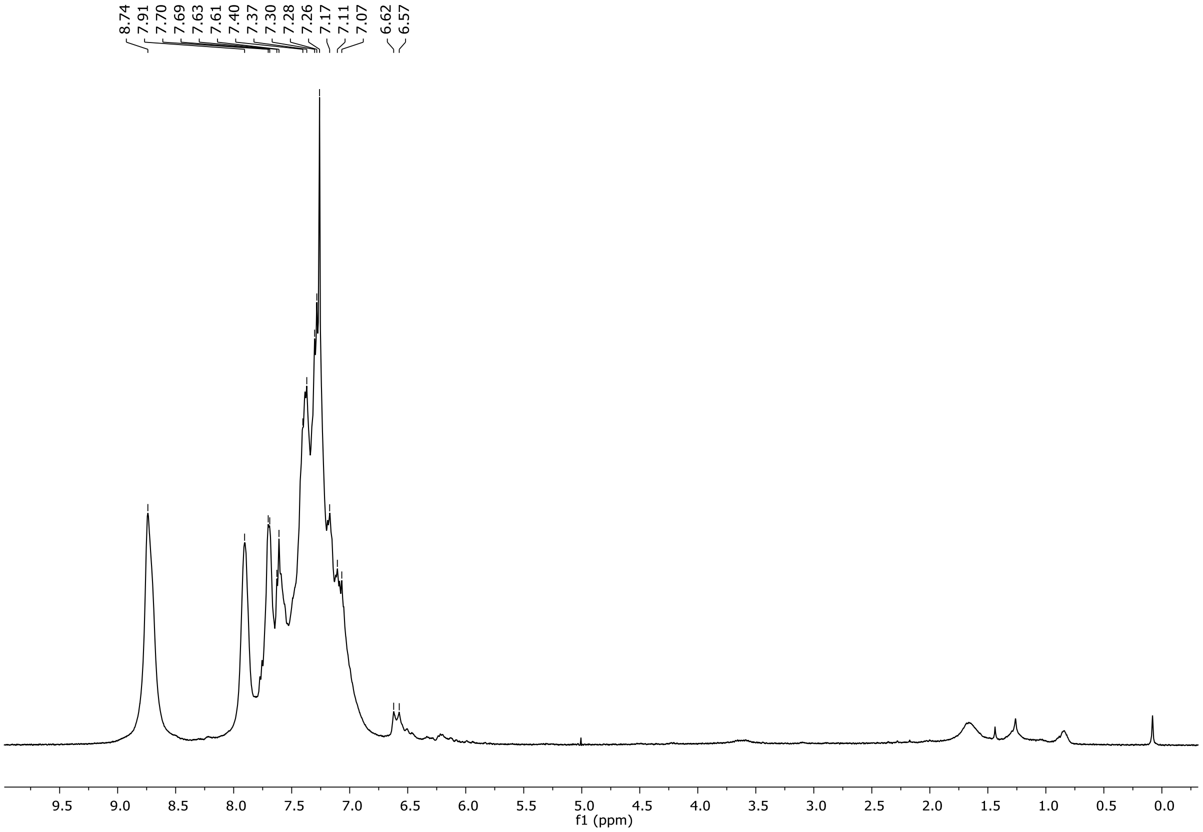

Figure S13. ^{1}H NMR spectra of DDSQ_Tb (400 MHz, $CDCl_3$)

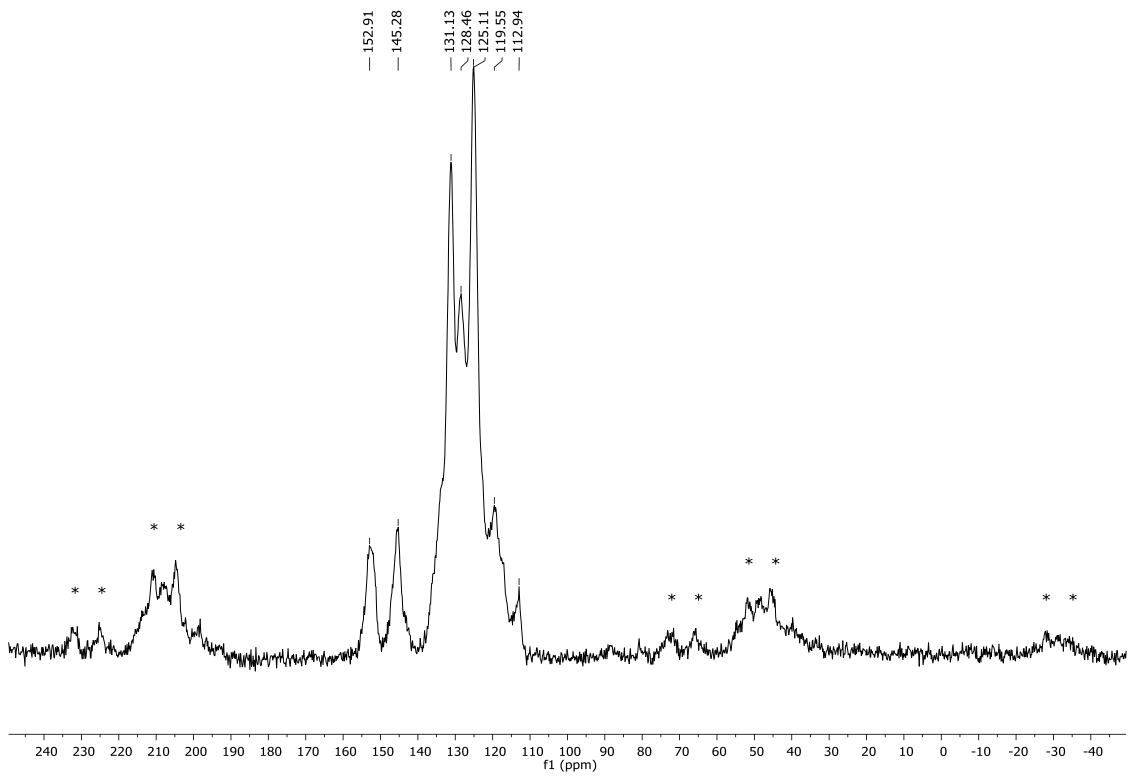

Figure S14. CP-MAS ^{13}C NMR spectra of DDSQ_Tb (125 MHz, 8kHz). Asterisks denote spinning sidebands.

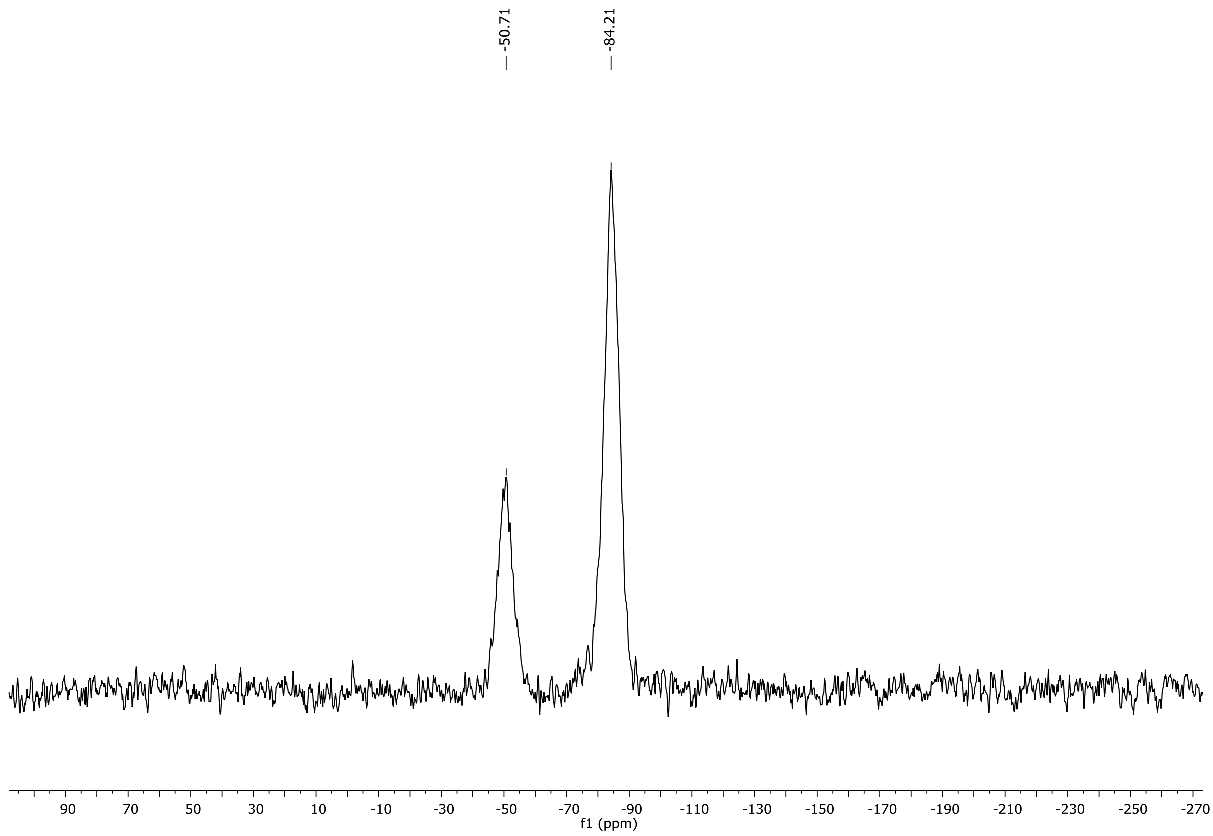

Figure S15. CP-MAS ^{29}Si NMR spectra of DDSQ_Tb (99.3 MHz, 8kHz).

3.1 Thermal analysis of reagents DDSQa-b and DDSQ_Ta-b

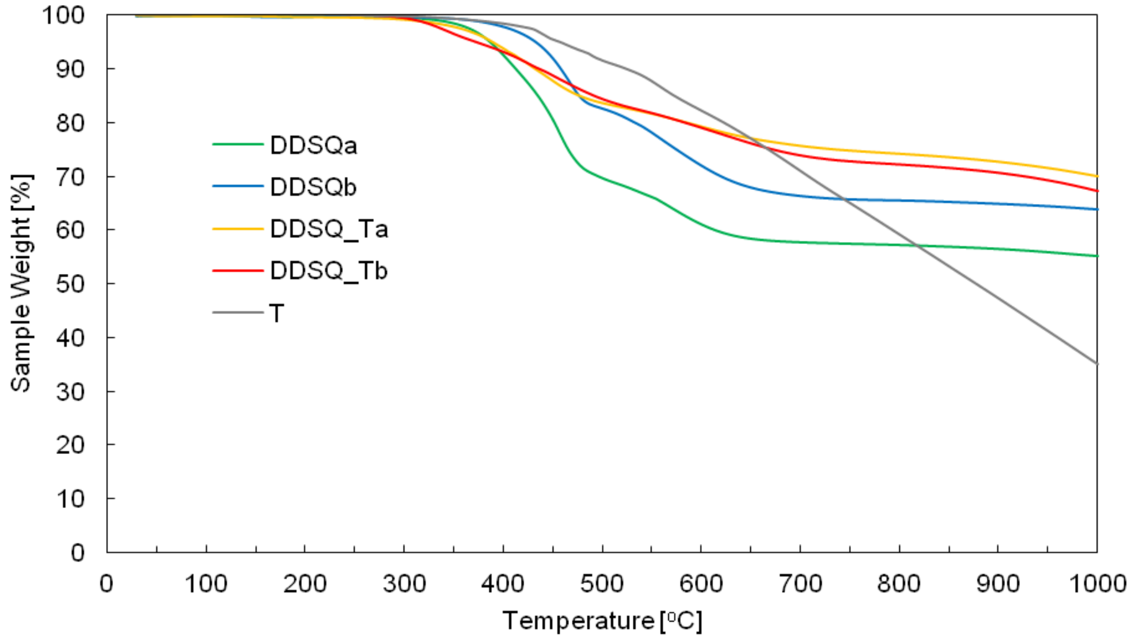

Figure S16. TGA analysis of reagents performed in nitrogen atmosphere.

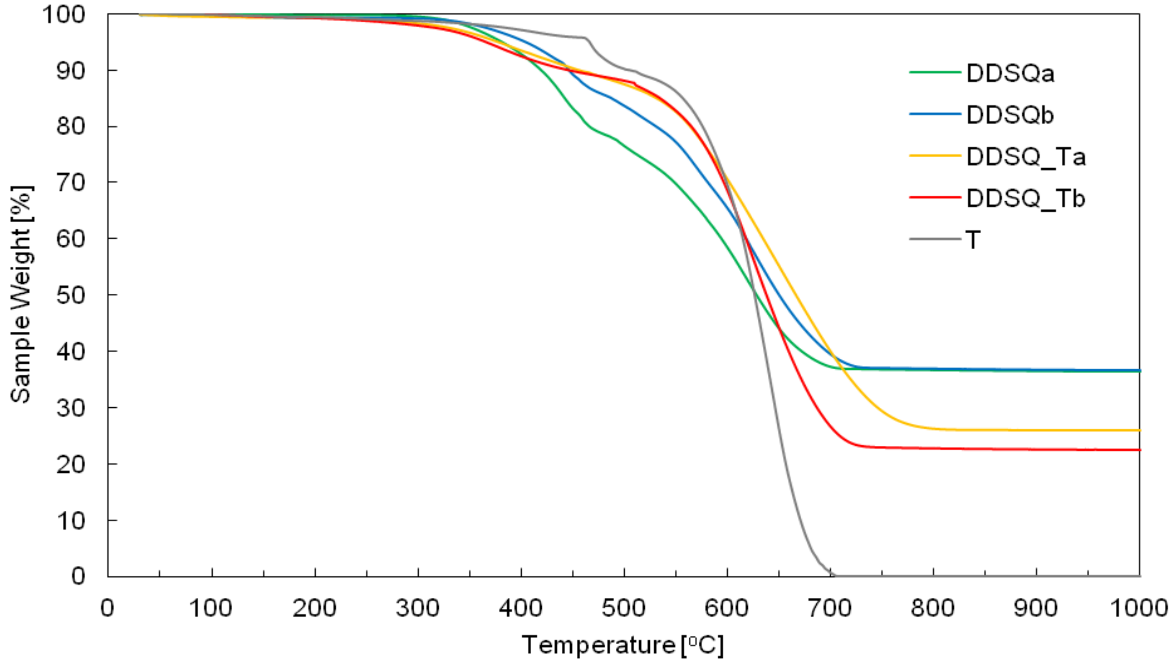

Figure S17. TGA analysis of reagents performed in air atmosphere.

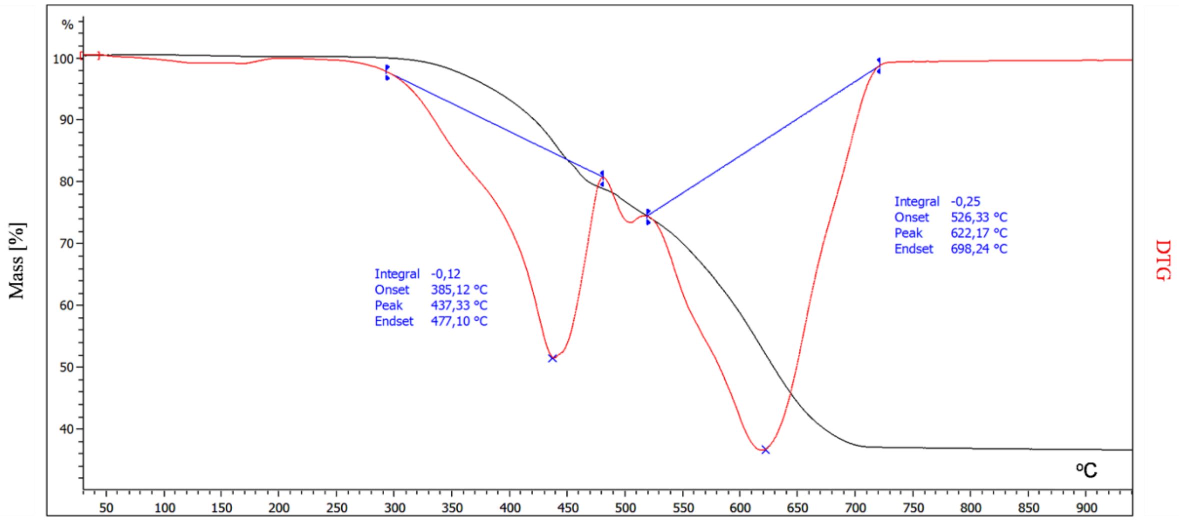

Figure S18. DTG curves of **DDSQa** in air atmosphere.

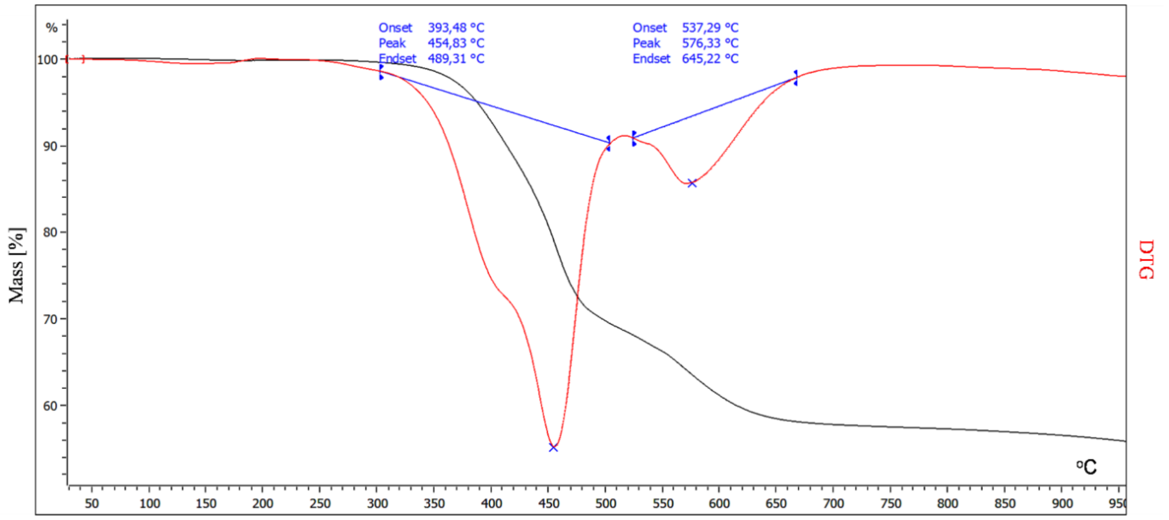

Figure S19. DTG curves of **DDSQa** in nitrogen atmosphere.

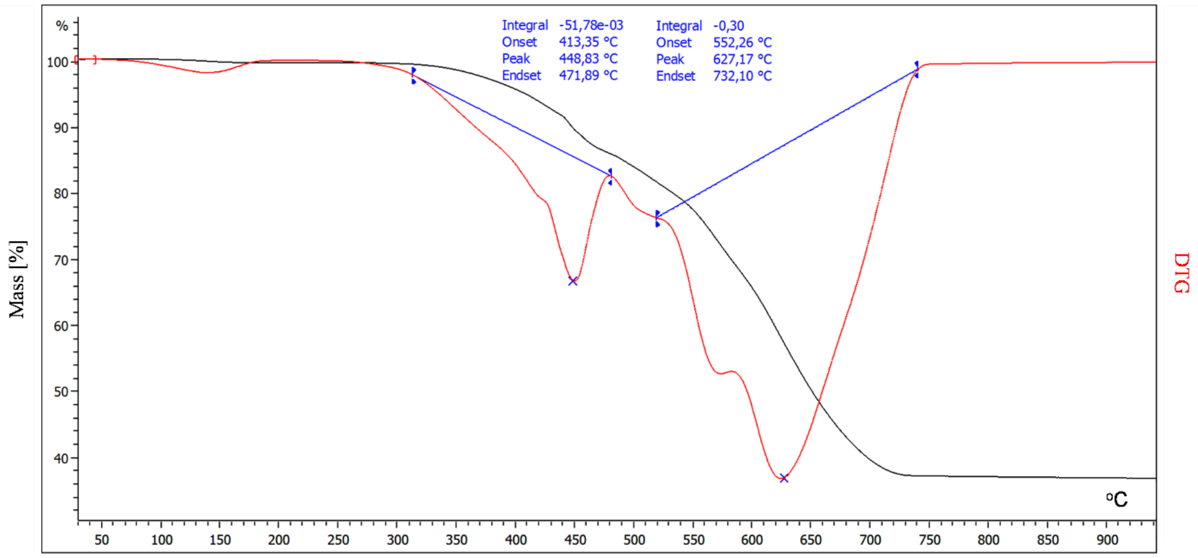

Figure S20. DTG curves of **DDSQb** in air atmosphere.

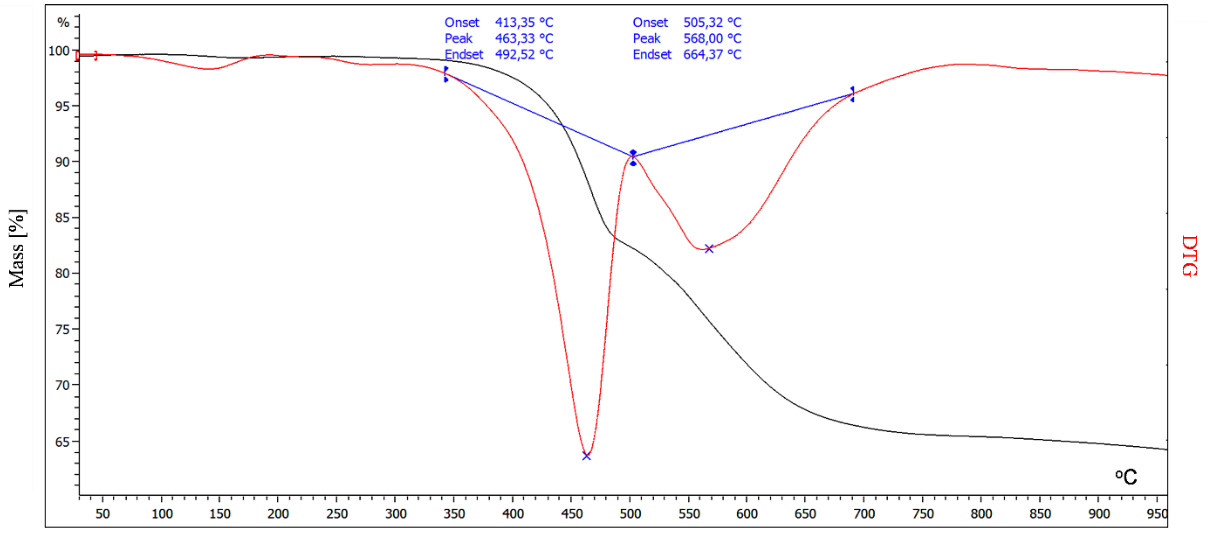

Figure S21. DTG curves of **DDSQb** in nitrogen atmosphere.

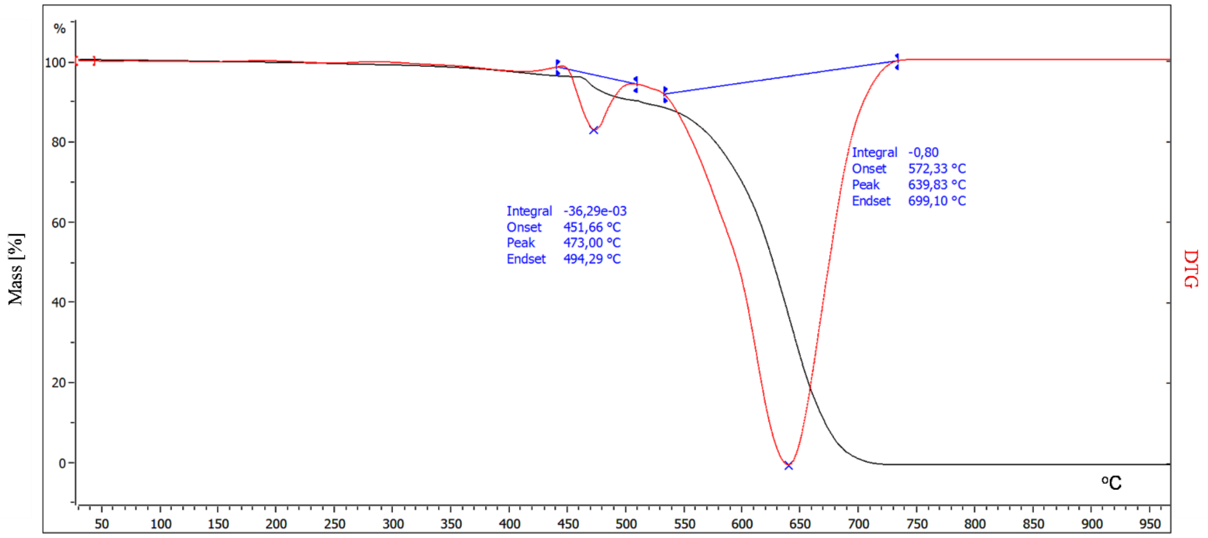

Figure S22. DTG curves of **T** in air atmosphere.

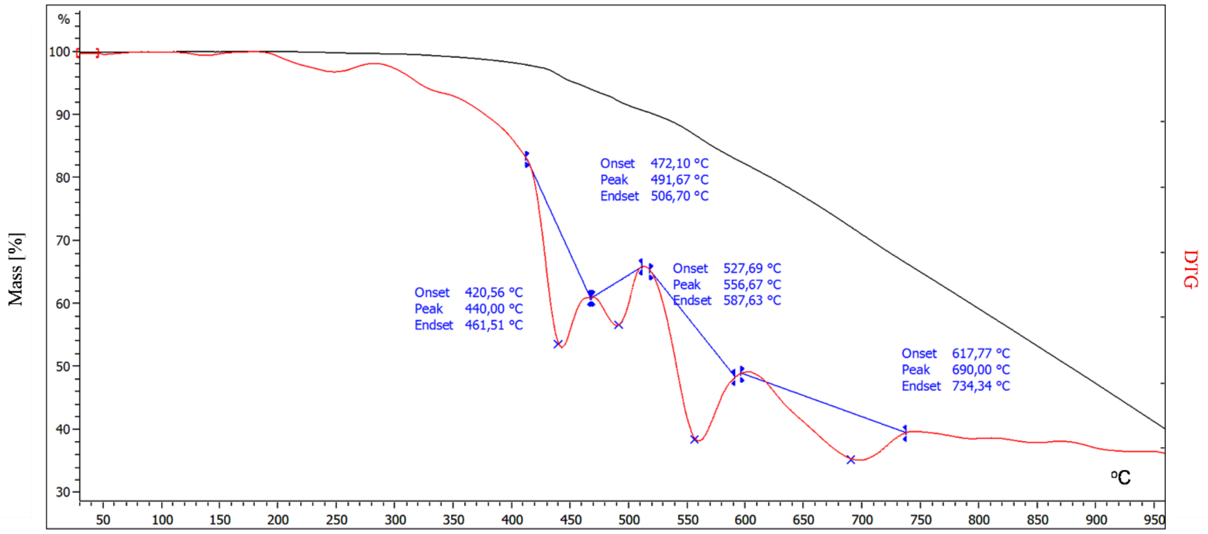

Figure S23. DTG curves of **T** in nitrogen atmosphere.

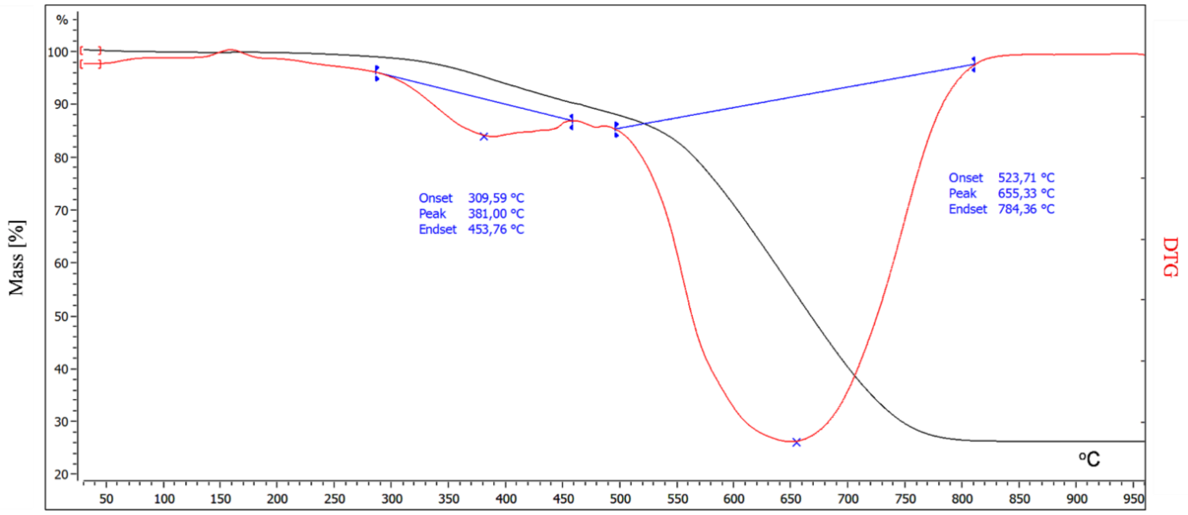

Figure S24. DTG curves of **DDSQ_Ta** in air atmosphere.

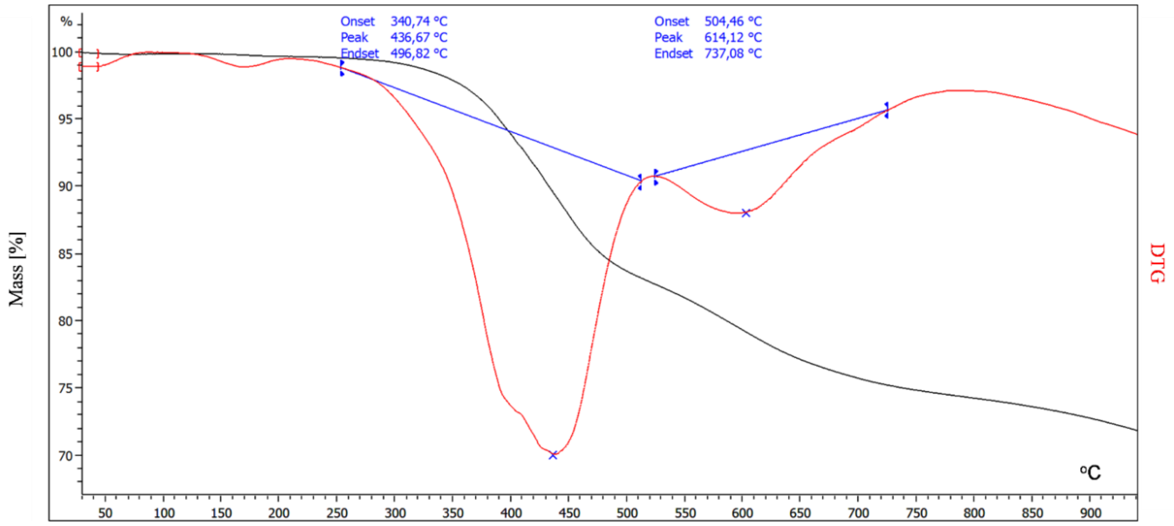

Figure S25. DTG curves of **DDSQ_Ta** in nitrogen atmosphere.

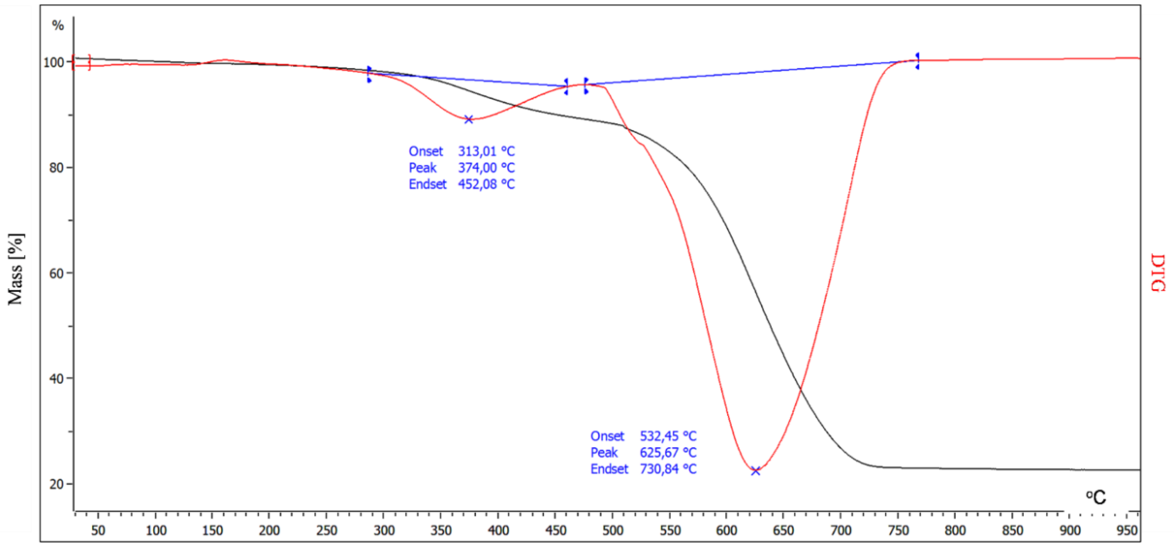

Figure S26. DTG curves of **DDSQ_Tb** in air atmosphere.

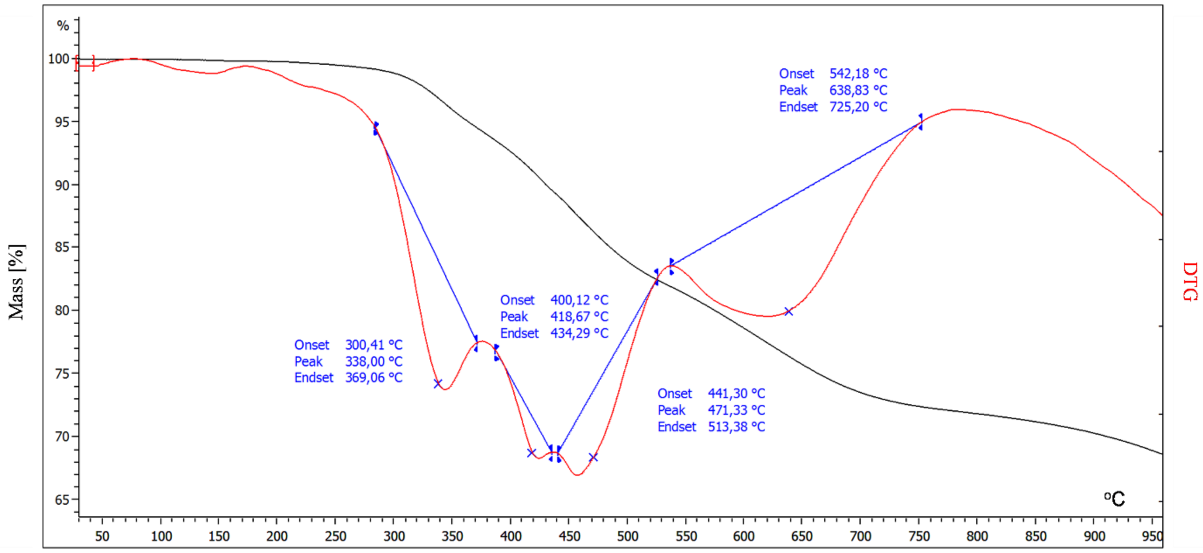

Figure S27. DTG curves of **DDSQ_Tb** in nitrogen atmosphere.

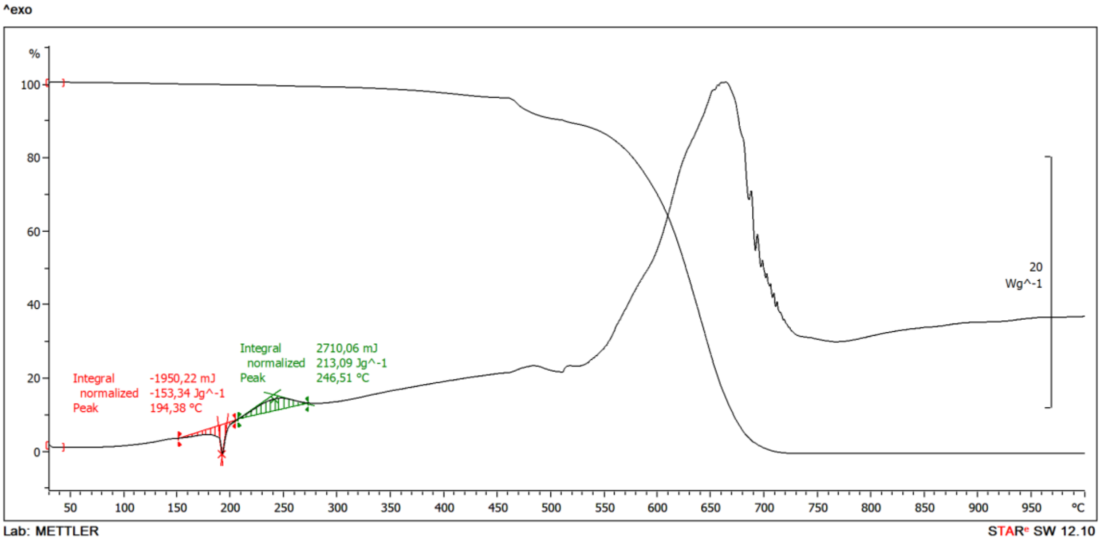

Figure S28. TGA/DSC curves of **T** in air atmosphere.

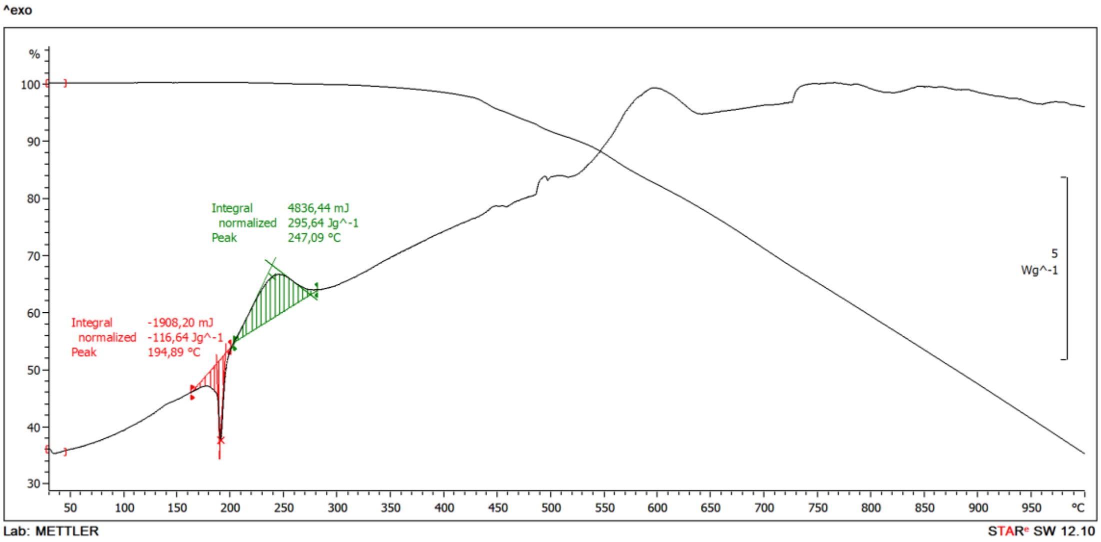

Figure S29. TGA/DSC curves of **T** in nitrogen atmosphere.

4. Results of absorption – emission analysis of DDSQa-b and DDSQ_Ta-b

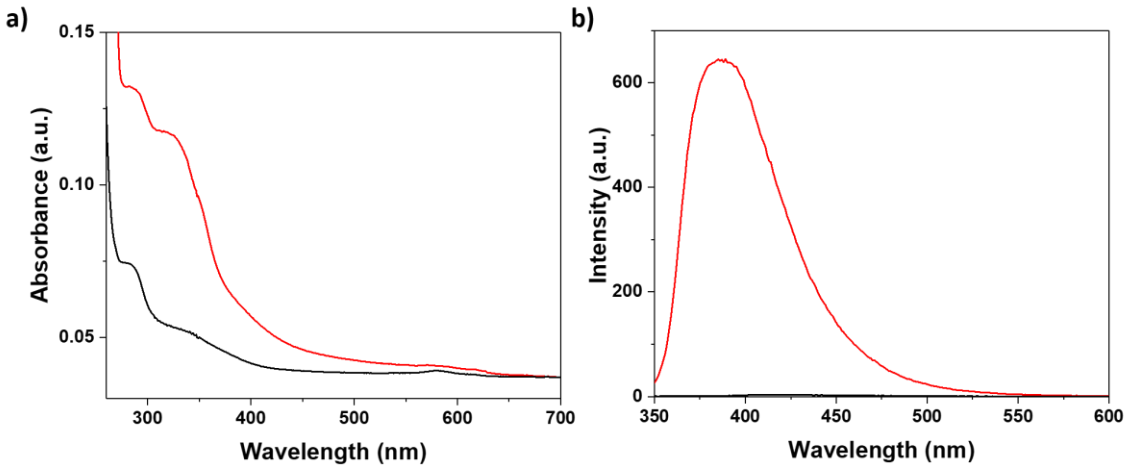

Figure S30. (a) Absorption and (b) emission spectra of the sample **DDSQa** (black) and the **DDSQ_Ta** (red) in CH_2Cl_2 (1×10^{-6} M). λ_{ex} = 310 nm, Slits = 5 nm.

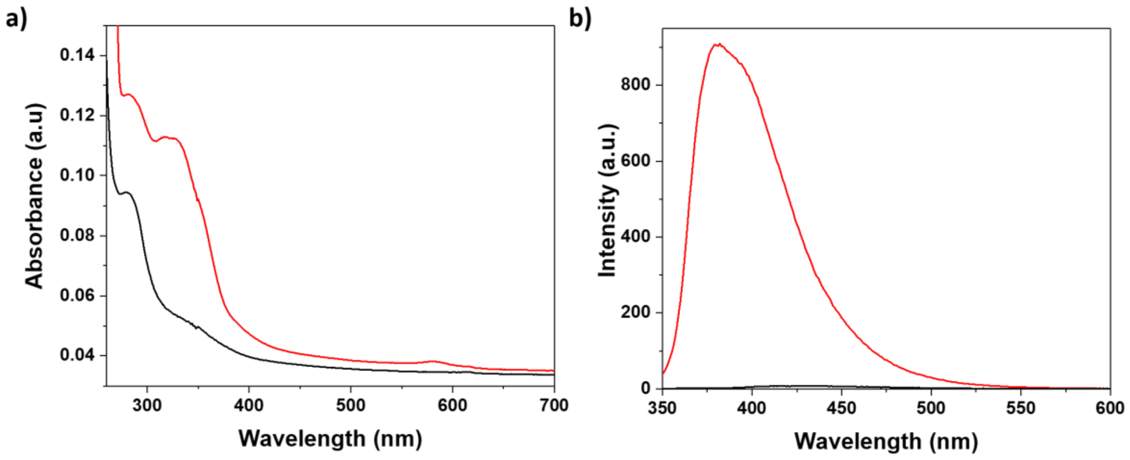

Figure S31. (a) Absorption and (b) emission spectra of the comparison of the compound **DDSQb** (black) and the **DDSQ_Tb** (red) in CH_2Cl_2 (1×10^{-6} M). λ_{ex} = 310 nm, Slits = 5 nm.

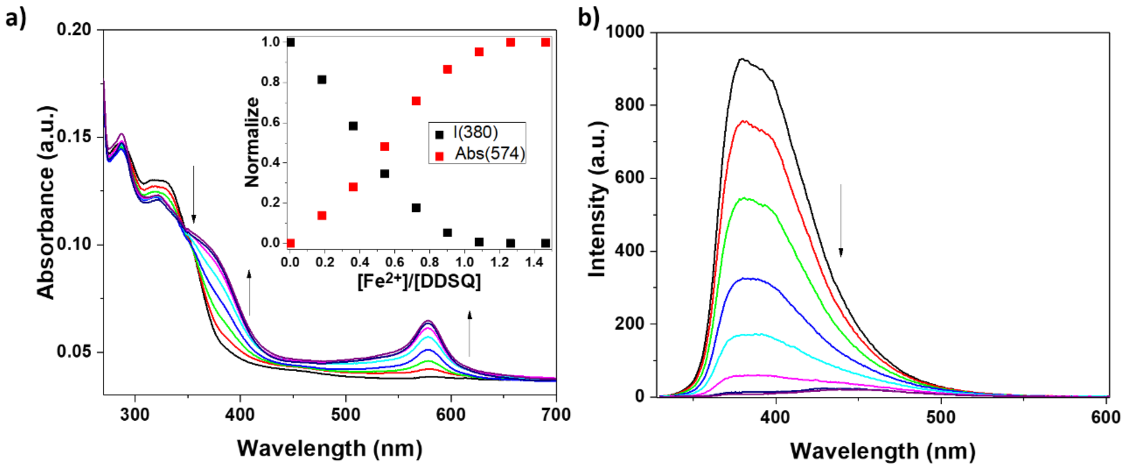

Figure S32. (a) UV-vis Absorption spectra of the sample **DDSQ_Tb** in CH_2Cl_2 (1×10^{-6} M) upon titration with $Fe(OTf)_2$ in EtOH (3.63×10^{-4} M). The inset shows the normalized absorption changes at 574 nm (red squares) and the normalized emission intensity changes at 380 nm (black squares). (b) Emission spectra of the compound **DDSQ_Tb** in CH_2Cl_2 (1×10^{-6} M) upon titration with $Fe(OTf)_2$ in EtOH (3.63×10^{-4} M). λ_{ex} = 310 nm, and OD = 0.13, Slits = 5 nm.

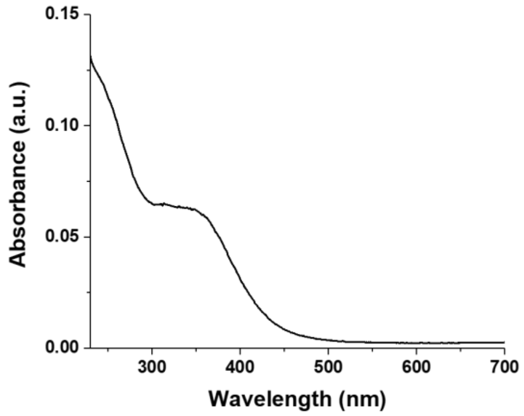

Figure S33. UV-vis Absorption spectra of $Fe(OTf)_2$ in EtOH (3.63×10^{-4} M).

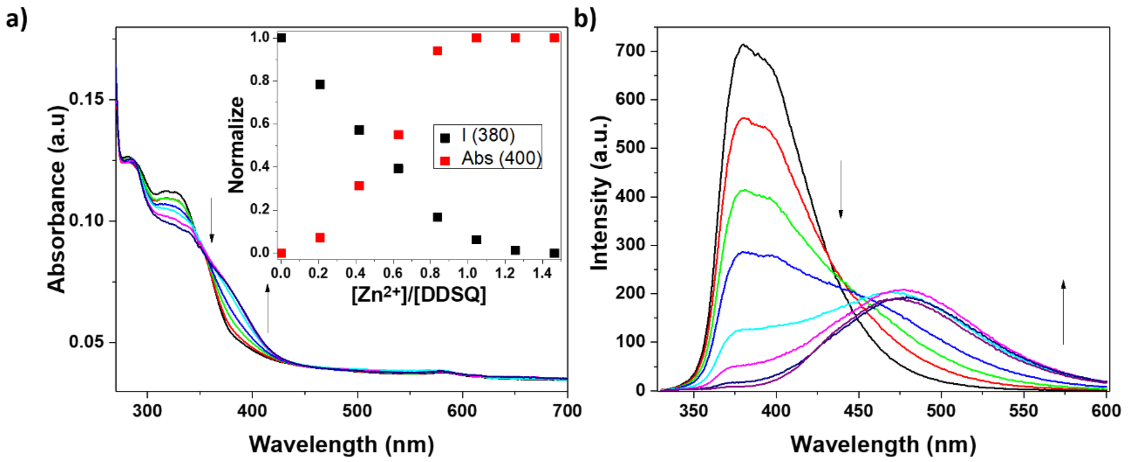

Figure S34. (a) UV-vis Absorption spectra of sample **DDSQ_Tb** in CH_2Cl_2 (1×10^{-6} M) upon titration with $Zn(OTf)_2$ in EtOH (4.18×10^{-4} M). The inset shows the normalized absorption changes at 400 nm (red squares) and the normalized emission intensity changes at 380 nm (black squares). (b) Emission spectra of the compound **DDSQ_Tb** in CH_2Cl_2 (1×10^{-6} M) upon titration with $Zn(OTf)_2$ in EtOH (4.18×10^{-4} M). λ_{ex} = 310 nm, and OD = 0.12, Slits = 5 nm.

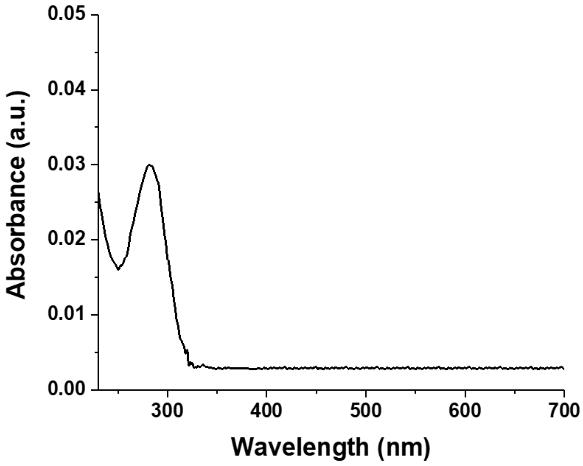

Figure S35. UV-vis Absorption spectra of $Zn(OTf)_2$ in EtOH (4.18×10^{-4} M).

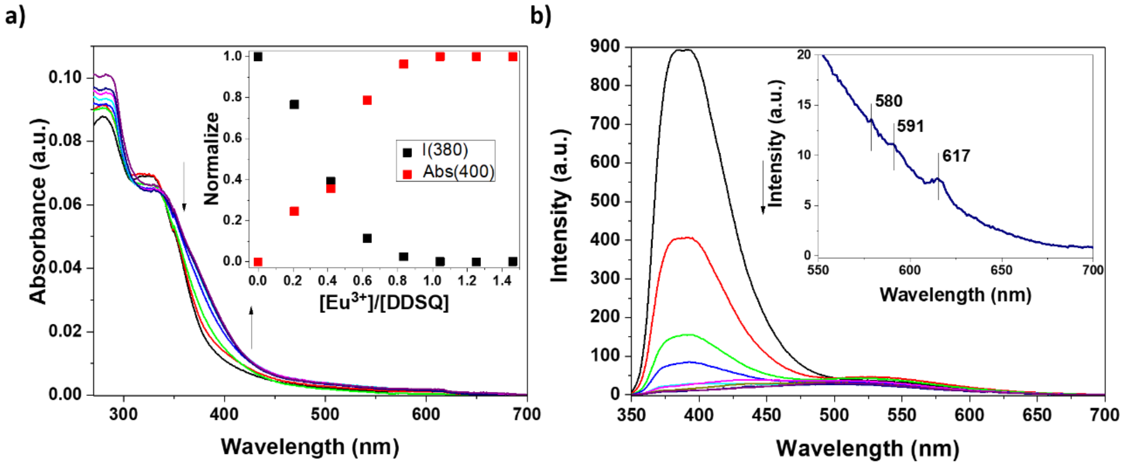

Figure S36. (a) UV-vis Absorption spectra of the compound **DDSQ_Tb** in CH_2Cl_2 (1×10^{-6} M) upon titration with $Eu(OTf)_3$ in EtOH (4.17×10^{-4} M). The inset shows the normalized absorption changes at 400 nm (red squares) and the normalized emission intensity changes at 380 nm (black squares). (b) Emission spectra of the compound **DDSQ_Tb** in CH_2Cl_2 (1×10^{-6} M) upon titration with $Eu(OTf)_3$ in EtOH (4.17×10^{-4} M). The inset shows the emission spectra in the range of 550 – 700 nm. λ_{ex} = 310 nm, and OD = 0.08, Slits = 5 nm.

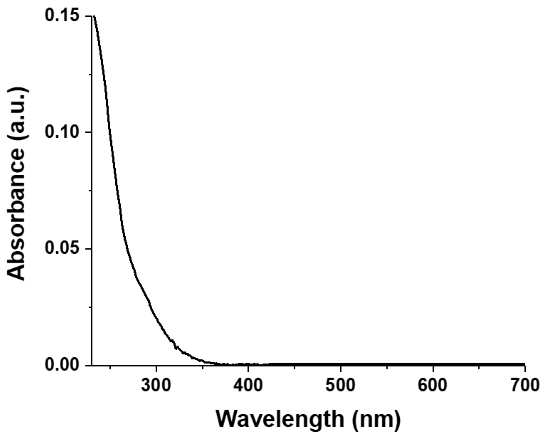

Figure S37. UV-vis Absorption spectra of $Eu(OTf)_3$ in EtOH (4.17×10^{-4} M).

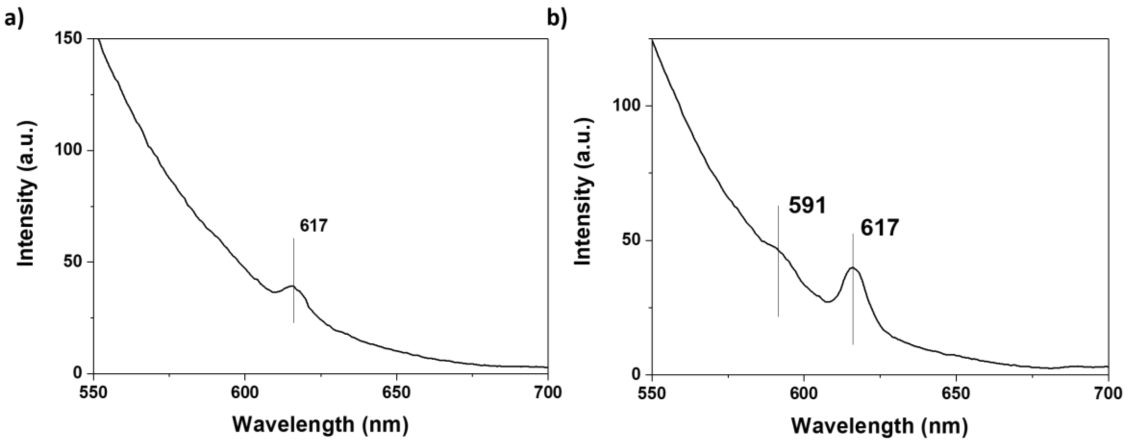

Figure S38. Typical Europium line like emission centred at 617 nm clearly visible in (a) compound **DDSQ_Ta** in CH_2Cl_2/CH_3CN (3/97) (1×10^{-6} M) upon titration with $Eu(OTf)_3$ in EtOH (4.17×10^{-4} M). (b) And compound **DDSQ_Tb** in CH_2Cl_2/CH_3CN (3/97) (1×10^{-6} M) upon titration with $Eu(OTf)_3$ in EtOH (4.17×10^{-4} M). Range of 550 – 700 nm. λ_{ex} = 310 nm, Slits =5 nm.

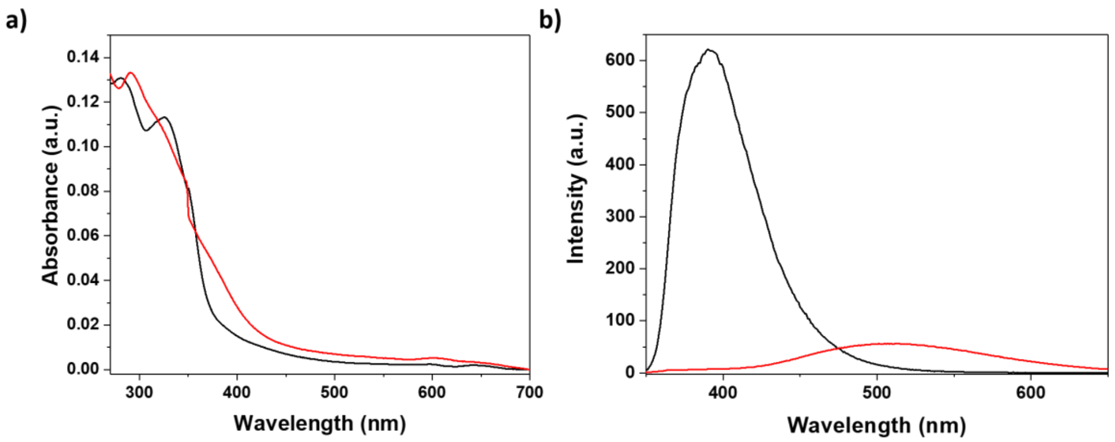

Figure S39. Absorption spectra and emission spectra of *E* (black line) to *Z* (red line) isomerization of the sample **DDSQ_Ta**. λ_{ex} = 310 nm, Slits = 5 nm.

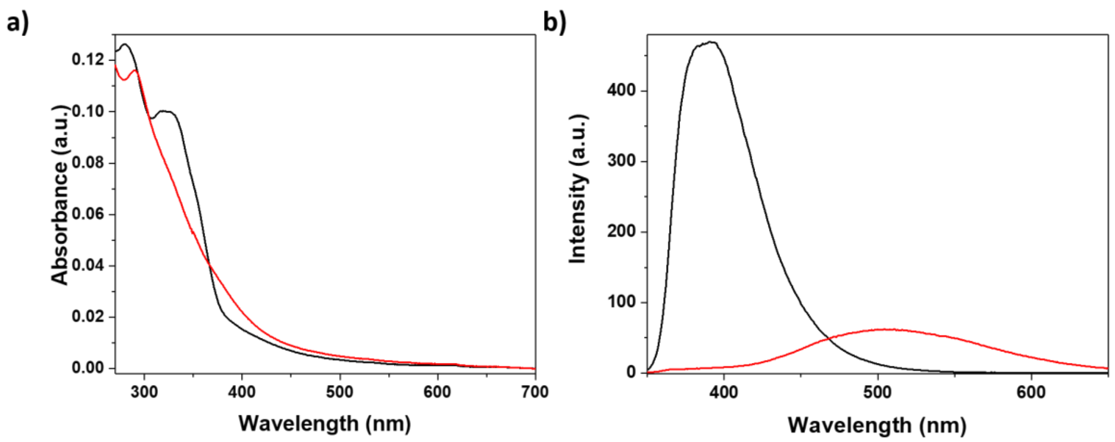

Figure S40. Absorption spectra and emission spectra of *E* (black line) to *Z* (red line) isomerization of the sample **DDSQ_Tb**. λ_{ex} = 310 nm, Slits = 5 nm**.**

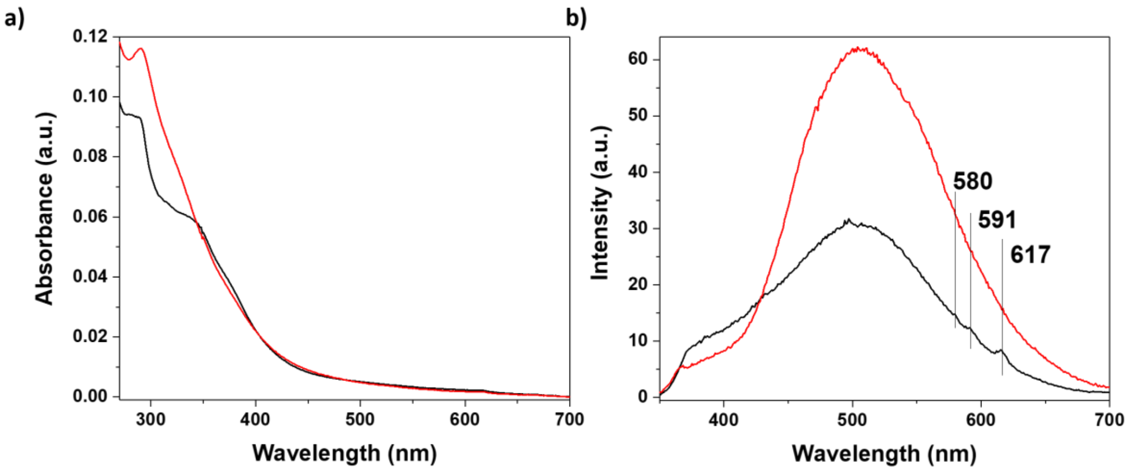

Figure S41. Absorption spectra and emission spectra **of Eu@DDSQ_Tb** *E* (black line) and *Z* (red line) isomers. λ_{ex} = 310 nm, Slits = 5 nm.

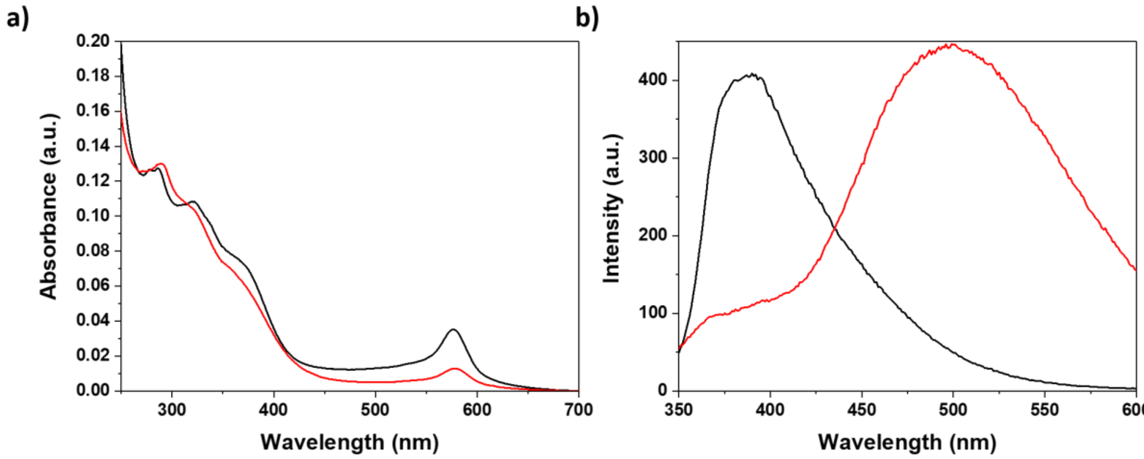

Figure S42. Absorption spectra and emission spectra of **Fe@DDSQ_Ta** *E* (black line) and *Z* (red line) isomers. λ_{ex} = 310 nm, Slits = 5 nm.

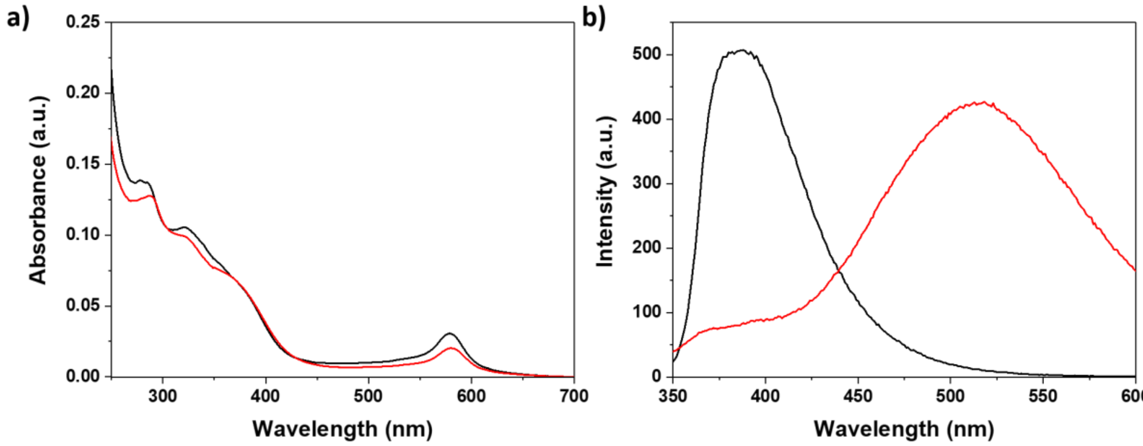

Figure S43. Absorption spectra and emission spectra of **Fe@DDSQ_Tb** E (black line) and Z (red line) isomers. λ_{ex} = 310 nm, Slits = 5 nm.

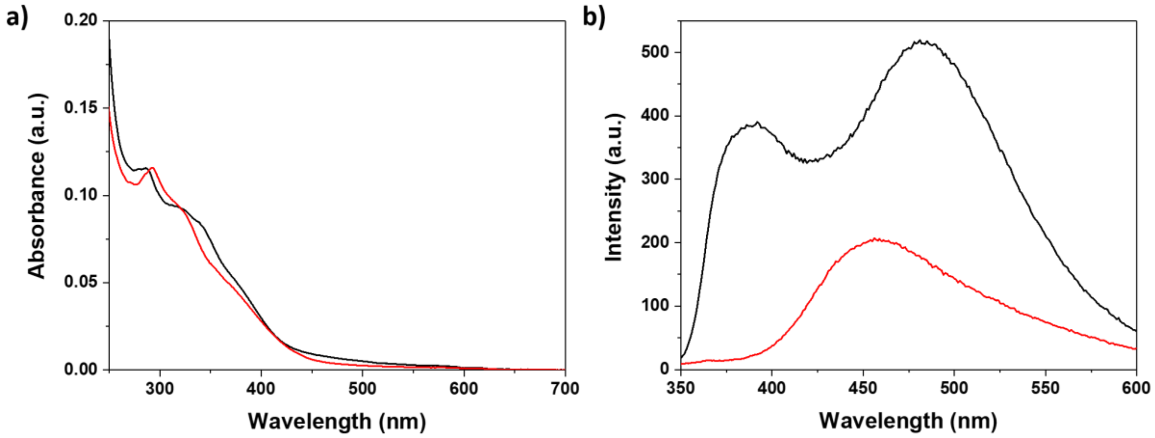

Figure S44. Absorption spectra and emission spectra of **Zn@DDSQ_Ta** *E* (black line) and *Z* (red line) isomers. λ_{ex} = 310 nm, Slits = 5 nm.

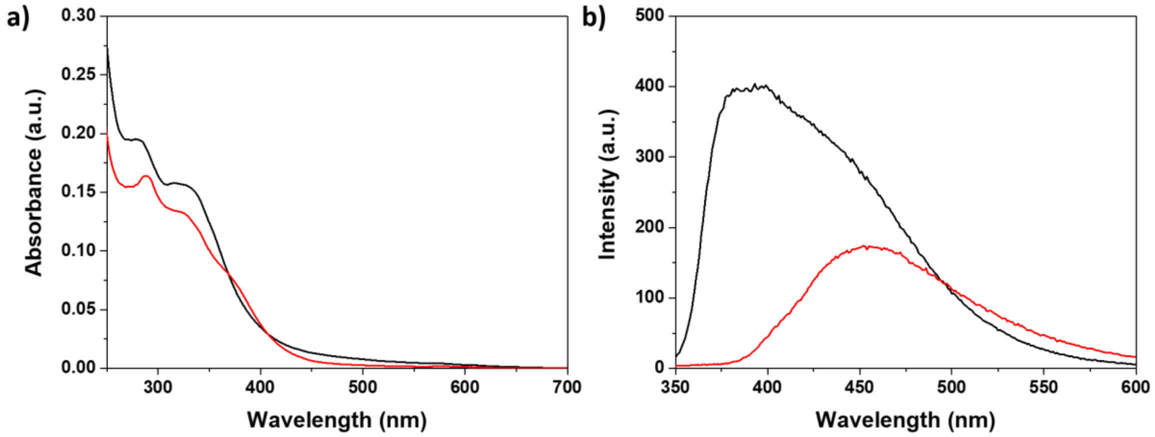

Figure S45. Absorption spectra and emission spectra of **Zn@DDSQ_Tb** *E* (black line) and Z (red line) isomers. λ_{ex} = 310 nm, Slits = 5 nm.

5. Results of analysis of DDSQa-b and DDSQ_Ta-b

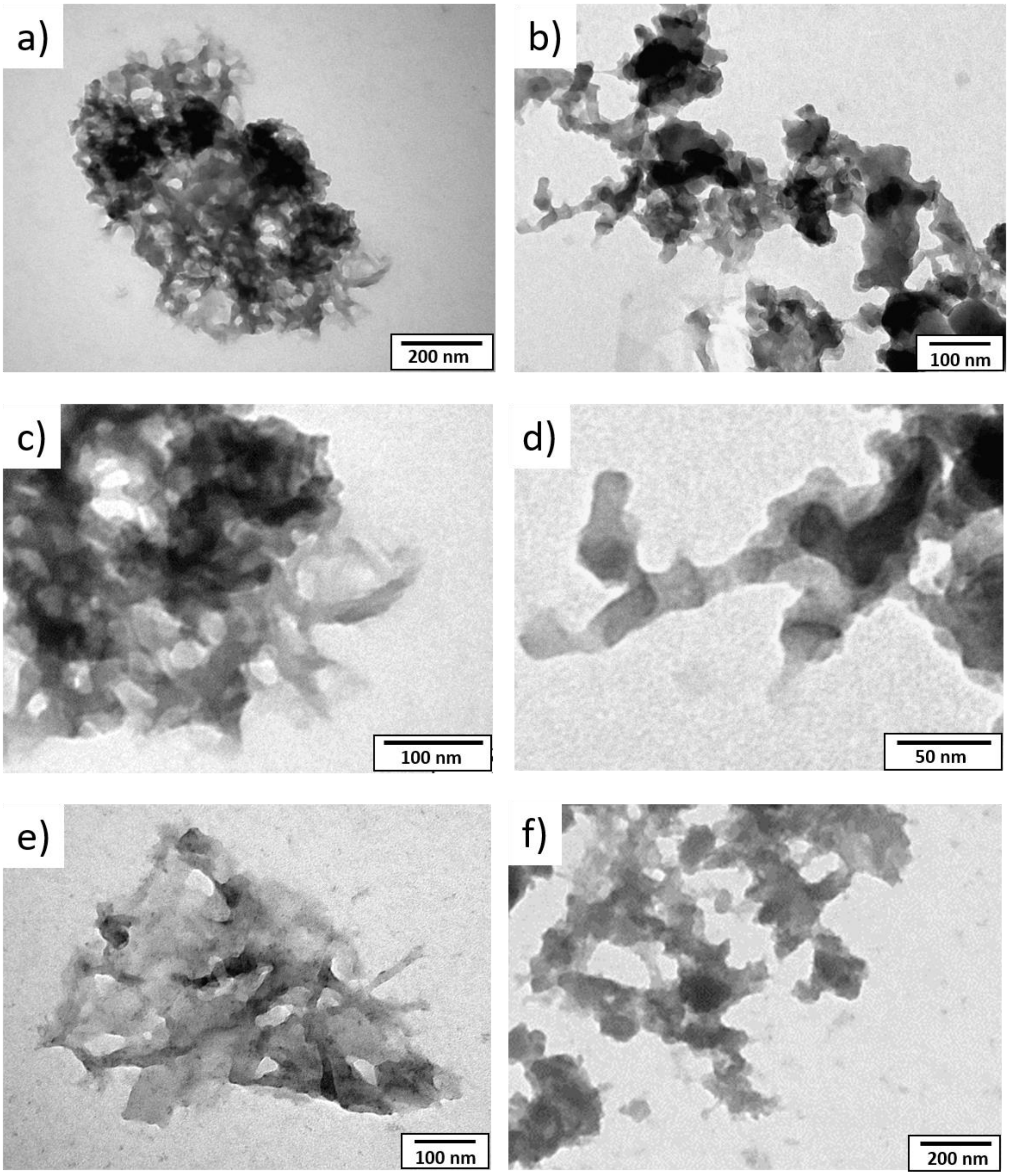

Figure S46. Transmission Electron Microscopy images of the lyophilized a) and c) **Zn@DDSQ_Ta**, b) and d) **Zn@DDSQ_Tb**, e) **2Eu@3DDSQ_Ta** and f) **2Eu@3DDSQ_Tb**.

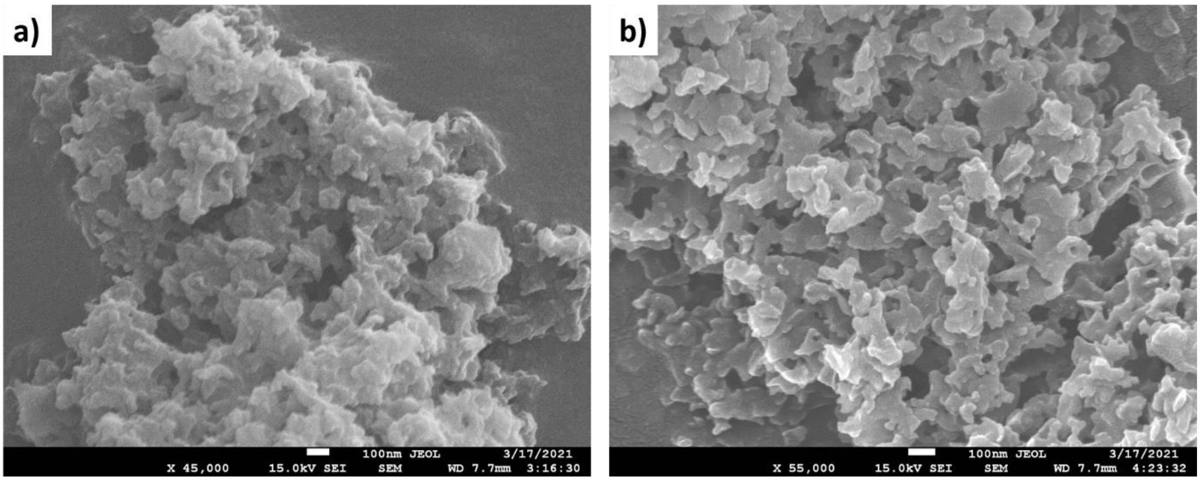

Figure S47. Scanning Electron Microscopy (SEM) images of the lyophilized (a) **Fe@DDSQ_Ta** and (b) **Fe@DDSQ_Tb**.

6. References

1 Winter, A.; Egbe, D. A. M.; Schubert, U. S. Rigid π-Conjugated Mono-, Bis-, and Tris(2,2':6',2"-terpyridines). *Org. Lett.* **2007**, 9, 2344–2348.

2 Żak, P.; Dudziec, B.; Kubicki, M.; Marciniec, B. Silylative Coupling versus Metathesis—Efficient Methods for the Synthesis of Difunctionalized Double-Decker Silsesquioxane Derivatives. *Chem. - A Eur. J.* **2014**, 20, 9387–9393.
